# Realizing highly efficient electrofluorescence through a co-axial hybrid local and charge-transfer (HLCT) excited state

**DOI:** 10.1039/d5sc06557g

**Published:** 2025-10-10

**Authors:** Kuo Yu, Yingbo Lv, Yilong Li, Zirui Wang, Shi-Tong Zhang, Jinbei Wei, Shanfeng Xue, Chenguang Wang, Bing Yang

**Affiliations:** a State Key Laboratory of Supramolecular Structure and Material, College of Chemistry, Jilin University Changchun 130012 P. R. China; b State Key Laboratory of Integrated Optoelectronics (JLU Region), College of Electronic Science and Engineering, Jilin University Changchun 130012 China; c Key Laboratory of Rubber-Plastics of the Ministry of Education, School of Polymer Science & Engineering, Qingdao University of Science and Technology 53 Zhengzhou Road Qingdao 266042 P. R. China stzhang@jlu.edu.cn jinbwei@jlu.edu.cn sfxue@qust.edu.cn

## Abstract

In this work, we report two high-performance donor–acceptor (D–A) electrofluorescent materials DPXZ-PI and DPXZ-PICN which are designed with novel co-axial hybrid local and charge-transfer (HLCT) excited states. The co-axial HLCT state features the direct bonding of a strong electron donor diphenoxazine (DPXZ) with a weak electron acceptor phenanthroimidazole (PI). This co-axial HLCT state is initially constructed in DPXZ-PI resulting in a high photoluminescence quantum yield (PLQY) of 81.7% and a high maximum external quantum efficiency (EQE) of 14.8% in a doped organic light-emitting diode (OLED), which is among the best results obtained for HLCT emitter-based OLEDs. Notably, the co-axial HLCT state is stable upon the introduction of the strong electron acceptor cyano-benzene, thus allowing an increase in the electron mobility in the OLED and resulting in a high EQE of 11.6% in a non-doped OLED based on DPXZ-PICN. Overall, the co-axial HLCT state facilitates an increase in the PLQY while maintaining exciton utilizing efficiency (EUE) in the OLED, and thus it is an effective instrument for producing highly-efficient electrofluorescent materials and devices.

## Abstract 2

Two highly efficient electrofluorescent materials with co-axial HLCT excited states are investigated. A maximum EQE of up to 14.8% is achieved due to the high PLQY of 81% and efficient triplet utilization in the OLEDs based on these materials.

## Graphical abstract

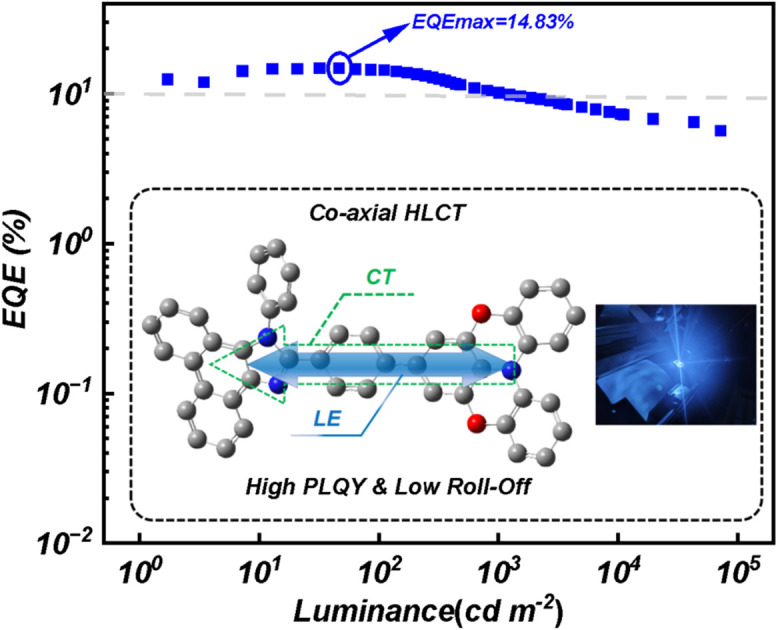

## 1 Introduction

Organic light-emitting diodes (OLED) represent a critical research direction in optoelectronic materials and devices due to their broad prospects for application in solid-state lighting, flexible displays and sensors. However, conventional fluorescent OLEDs can only utilize the electrically generated singlet excitons (S_1_) which represent 25% of the excitons generated, while the other 75% of excitons, the triplet excitons (T_1_), are dissipated *via* non-radiative decay.^1^ To circumvent this fundamental spin-statistics limit and achieve a 100% exciton utilization efficiency (EUE) in low-cost electro-fluorescence devices, researchers have proposed diverse exciton harvesting strategies, including triplet-triplet annihilation (TTA),^2–6^ thermally activated delayed fluorescence (TADF),^7–31^ hybrid local and charge transfer (HLCT)^32–47^ and stable doublet states. In particular, charge transfer (CT) excited states have a positive influence on triplet harvesting, because the CT state can reduce the singlet-triplet energy gap by lowering the degree of orbital overlap, thereby completely converting the electro-generated triplet excitons into singlet excitons. Furthermore, an adequate spin–orbit coupling (SOC) coefficient can accelerate the reversed intersystem crossing (RISC) by the incorporation of an LE component to adapt to the parity selection rule for RISC (known as the El-Sayed rule). This approach is becoming the basic principle of TADF and HLCT.^48,49^ The HLCT mechanism offers distinct advantages over the TADF mechanism, such as short excited-state lifetimes, suppressed efficiency roll-off and high external quantum efficiency (EQE) in non-doped devices. In 2012, Li *et al.* reported the first blue-emitting HLCT material based on a triphenylamine-phenanthroimidazole (TPA-PPI) framework, achieving a breakthrough beyond the spin-statistics limit, however, the realized EUE remained limited to merely 28% due to the relatively weak CT character.^32^ Subsequently, in 2015, Zhang *et al.* significantly advanced this approach. By introducing a perpendicular cyano group substituent onto the TPA-PPI backbone, they engineered an orthogonal HLCT construction with strong CT character. This modification dramatically increased the EUE to nearly 98%, concurrently demonstrating that the CT component within the HLCT state is crucial for the complete harvesting of electrically generated triplets. The resulting OLED achieved a record-setting EQE of 7.8%, representing the highest reported EQE for non-doped blue OLEDs at that time.^47^ Most recently, a pioneering work by Tang and Wang *et al.* further demonstrated the superior properties and potential of orthogonal HLCT materials by constructing a cross-axis configuration which achieved high-performance multifunctional OLEDs.^50^ However, orthogonal HLCT systems still have problems in terms of the strong CT character, which can lead to a significant reduction in PLQY due to its intrinsically low orbital overlap, thereby fundamentally limiting the upper bound of the EQE according to the following relation.EQE = *η*_e−h_·PLQY·*η*_out_·EUEHere, *η*_e−h_represents the exciton recombination efficiency and in structurally optimized OLEDs this value should ideally approach 100% and *η*_out_ represents the light-outcoupling efficiency, which is estimated to be 20–30% in a glass-substrate bottom-emissive OLED depending on the micro-structural regularity of the functional layers in the OLED. For example, the above mentioned orthogonal HLCT material TBPMCN exhibits a PLQY of merely 40%, and the upper-limit of the EQE is only 8% when *η*_out_ is estimated to be 20%. Therefore, an innovative molecular design that can substantially enhance the PLQY while preserving the required CT properties for full EUE is needed.

In this work, we employ a direct linkage between a strong electron donor diphenoxazine (DPXZ) and a weak electron acceptor phenanthroimidazole (PI) to ensure that the CT transition and LE transition are in the same direction, and to construct a novel co-axial HLCT excited state. According to this molecular design, two highly efficient emitters DPXZ-PI and DPXZ-PICN were synthesized and investigated. The co-axial HLCT materials exhibit PLQYs nearly double those of orthogonal HLCT materials, thus boosting the EQE to 14.8% (doped OLED based on DPXZ-PI) and 11.6% (non-doped OLED based on DPXZ-PICN). This comparative result unequivocally demonstrates that the co-axial molecular design strategy constitutes a novel approach for achieving high exciton utilization *via* a CT state while simultaneously realizing a significantly boosted PLQY, ultimately leading to an exceptional EQE.

## 2 Results and discussions

### 2.1 Molecular design

**Fig. 1:**
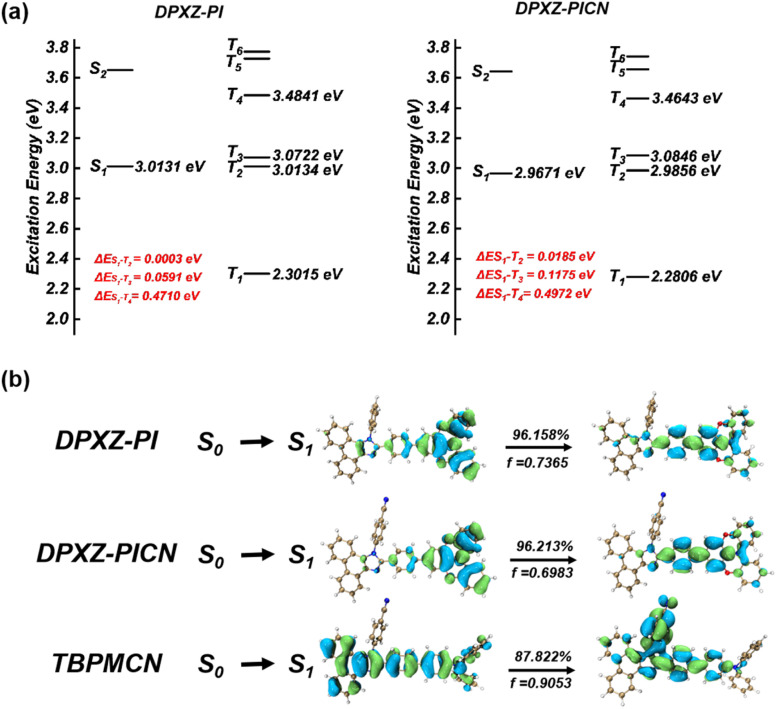
(a) Excitation energy diagrams of DPXZ-PI and DPXZ-PICN; (b) S_0_ → S_1_ NTO of DPXZ-PI, DPXZ-PICN and TBPMCN. The geometry optimization and excited state estimation were carried out using the Gaussian 16 A.03 package^55^ at the m062x/6-31g (d, p) and td-m062x/6-31g (d, p) levels of theory, respectively. The natural transition orbital (NTO) images were generated using the MultiWFN 3.8 dev package.^56^ The SOC coefficient was calculated using the beijing density-functional (BDF) package at the td-m062x/6-31g (d, p) level of theory.^57–60^

**Scheme 1:**
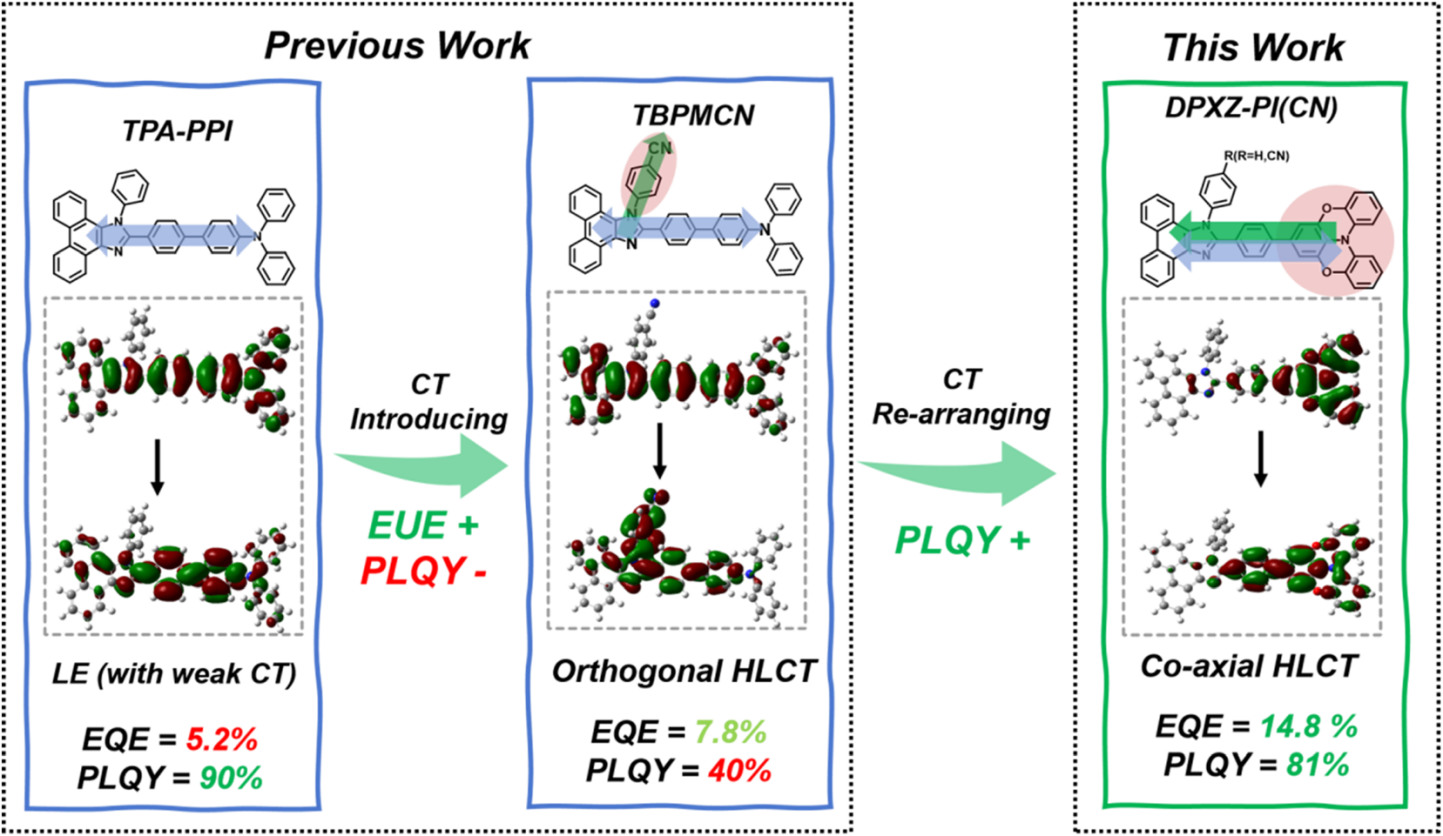
Schematic representation of the excited state design, photophysical performance and OLED performance of the weak-CT HLCT, orthogonal HLCT and co-axial HLCT materials.

To illustrate the excited state properties of the co-axial HLCT state, we first carried out density-functional theory (DFT) and time-dependent DFT (TDDFT) calculations at the m062x/6-31g (d, p) level of theory to obtain the optimized geometries of the S_0_ and S_1_ states of DPXZ-PI and DPXZ-PICN along with their excitation energies and natural transition orbitals (NTOs) (Fig. 1). As depicted in Scheme 1, the orthogonal HLCT material TBPMCN shows two distinguishable transitions with an orthogonal distribution: the LE-like transition on the triphenylamine (TPA)-PI backbone (which is similar to that of TPA-PPI), and the CT-like transition from the TPA-PI backbone donor to the perpendicular cyano-benzene acceptor. In the cases of DPXZ-PI and DPXZ-PICN, the combination of the strong electron-donating ability of the DPXZ moiety and the relatively weak electron-accepting ability of PI firstly results in pronounced CT transition character from the DPXZ moiety to the imidazole ring. Spatial overlap between the S_1_ NTO “hole” and “electron” of DPXZ-PI and DPXZ-PICN can be observed clearly within the DPXZ donor and the adjacent phenyl ring, which is consistent with the relatively high oscillator strength of the S_1_ excited states (*f* = 0.7365 for DPXZ-PI and *f* = 0.6983 for DPXZ-PICN). Although the character of this transition seems to be similar to the previously reported HLCT material TPA-PPI (Scheme 1), which has relatively weak CT character, the strong donor ability of DPXZ in DPXZ-PI and DPXZ-PICN involves a greater *n* → π* transition or CT transition component, which accounts for the decreased oscillator strength of DPXZ-PI and DPXZ-PICN compared to TPA-PPI. More importantly, the stronger CT character in DPXZ-PI and DPXZ-PICN will no doubt lead to more efficient triplet harvesting. As shown in Fig. 1a, the T_3_ and T_2_ energy levels of DPXZ-PI and DPXZ-PICN are close to their S_1_ energy level, and large energy gaps of >0.7 eV are observed between the T_2_ and T_1_ energy levels, forming a typical “hot-exciton” channel. Furthermore, the SOC coefficients between the S_1_ and T_3_ energy levels of DPXZ-PI and DPXZ-PICZ are all large enough (0.61 cm^−1^ and 0.48 cm^−1^ for DPXZ-PI and DPXZ-PICN, respectively, Table 1) for high-lying reversed intersystem crossing (hRISC) to occur. This result obeys the El-Sayed rule that states that RISC is more efficient for the transition from *n* → π* states to π → π* states (Fig. S2)^51–54^ because the HLCT properties of the S_1_ excited states of DPXZ-PI and DPXZ-PICN have an LE component that can provide specific π → π* transition properties. Strikingly, different from our previous works,^32,47^ cyano-substitution of the phenyl side-group of DPXZ-PI (yielding DPXZ-PICN) results in no significant change in its NTO, revealing that the co-axial HLCT is robust upon substitution of the strong acceptor, thus potentially facilitating electron injection and transportation in an OLED without severe PLQY reduction.

**Table 1:** SOC coefficients related to the S_1_ states of DPXZ-PI and DPXZ-PICN

	DPXZ-PI	DPXZ-PICN
S_1_-T_1_	0.10 cm^−1^	0.09 cm^−1^
S_1_-T_2_	0.12 cm^−1^	0.19 cm^−1^
S_1_-T_3_	0.61 cm^−1^	0.48 cm^−1^
S_1_-T_4_	0.22 cm^−1^	0.30 cm^−1^
S_1_-T_5_	0.29 cm^−1^	0.45 cm^−1^
S_1_-T_6_	0.54 cm^−1^	0.36 cm^−1^

**Fig. 2:**
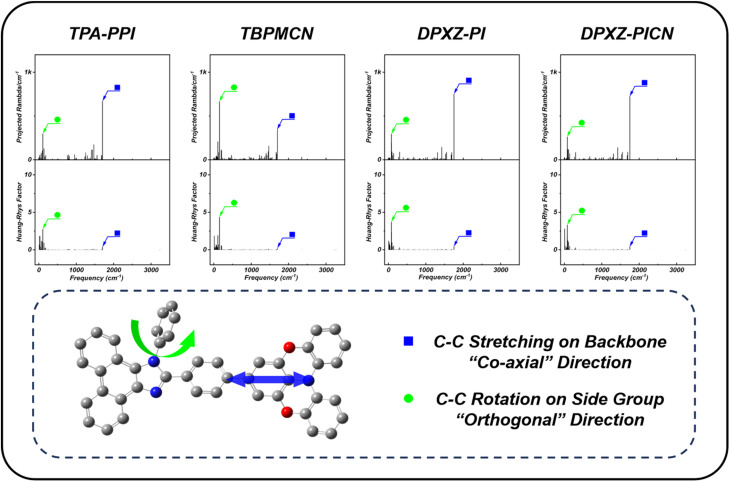
Projected re-organization energies, Huang–Rhys factors and corresponding vibrational modes of TPA-PPI, TBPMCN, DPXZ-PI and DPXZ-PICN. The calculations were carried out using the Gaussian 16 A.03 package and a molecular materials property prediction package (MOMAP).^61–66^

In addition to the radiative decay properties, the non-radiative decay rate constitutes another critical factor governing the PLQY of materials. Consequently, during frequency calculations, we further analyzed the reorganization energy projections for all normal vibrational modes as well as the Huang–Rhys factors (HR factors or S) for both DPXZ-PI and DPXZ-PICN. For comparative analysis, identical calculations were performed on the two reported benchmark structures in Scheme 1, that is, the weak-CT structure TPA-PPI and the strong-CT structure TBPMCN. Our analysis reveals that while the vibrational modes contributing significantly to the total reorganization energy exhibit similar distributions across all four materials, their relative strengths show distinct differences. As illustrated in Fig. 2, the dominant contribution to the reorganization energy of the orthogonally directed HLCT material TBPMCN arises from a low-frequency mode at 151.95 cm^−1^. This mode corresponds to the torsional vibration of the peripheral benzonitrile substituent relative to the TPA-PPI conjugated core. In stark contrast, the largest contributions to the reorganization energy of the weak-CT or the co-axially directed HLCT structures (TPA-PPI, DPXZ-PI and DPXZ-PICN) occur at higher frequencies (TPA-PPI: 1702.75 cm^−1^; DPXZ-PI: 1752.95 cm^−1^; DPXZ-PICN: 1751.03 cm^−1^). These modes primarily involve the C–C stretching vibrations of the TPA-PI or DPXZ-PI backbone. This disparity aligns fundamentally with the distinct HLCT architectures of the molecules: the vibrational modes dominating the reorganization energy are intrinsically linked to the D–A connectivity and the specific composition of the HLCT state. These results tie in with the similarity of the NTOs obtained for DPXZ-PI and DPXZ-PICN as well as with the CT components calculated in different directions (Table S1), where the CT ratios of DPXZ-PI and DPXZ-PICN in the co-axial direction are obviously larger than those in the orthogonal direction. Critically, these HLCT architecture-dependent vibrational modes directly govern their respective non-radiative decay rates. As shown in Fig. 2, the HR factor associated with the key C–C twisting mode at 151.95 cm^−1^ in TBPMCN is substantial (*S* = 4.38173). Conversely, the HR factors of the analogous high-frequency modes in the other three materials are orders of magnitude lower, demonstrating their significantly suppressed non-radiative decay rates compared to the strong CT structure TBPMCN. Consequently, even if the oscillator strength of the co-axial HLCT state is slightly lower, its inhibited non-radiative pathways enable the realization of high luminescence efficiency from the excited state. Furthermore, the DPXZ structure has two ether oxygen bridges which effectively suppress the phenyl ring vibrations when compared with TPA, thus contributing positively to the reduction of non-radiative decay. Finally, a comparison of the total reorganization energies of the four materials (TBPMCN: 3356.87 cm^−1^; TPA-PPI: 2929.56 cm^−1^; DPXZ-PI: 2739.20 cm^−1^; DPXZ-PICN: 2763.67 cm^−1^) shows that the strong CT structure TBPMCN exhibits the highest total reorganization energy, and the co-axial HLCT materials DPXZ-PI, DPXZ-PICN possess total reorganization energies that are even lower than the weak-CT structure TPA-PPI. Overall, the co-axial HLCT materials can facilitate higher PLQY due to their suppressed non-radiative transition profiles. Last but not least, in contrast to the large change in properties upon cyano-substitution of TPA-PPI to give TBPMCN, the cyano-substitution of DPXZ-PI does not makes any obvious difference to the dominate vibrational modes, which is also evidence that the co-axial HLCT remains stable upon functional group modification.

### 2.2 Excited state properties

**Fig. 3:**
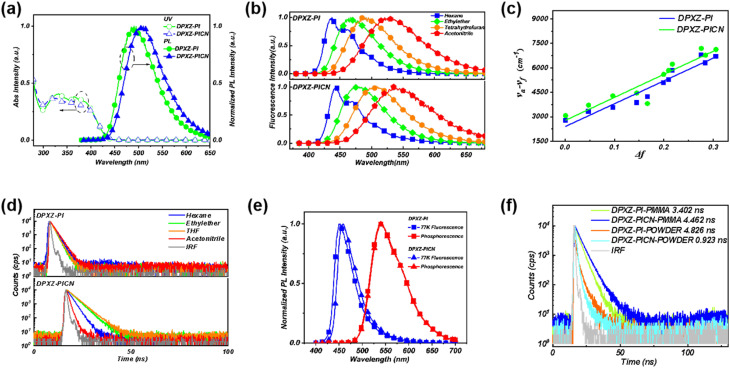
Photophysical properties of DPXZ-PI and DPXZ-PICN. (a) Absorption and emission spectra in dilute THF solution. (b) Solvatochromic emission spectra in dilute hexane, ethyl ether, THF and acetonitrile solutions. (c) Lippert–Mataga solvatochromic model. (d) Nanosecond-level lifetimes in dilute hexane, ethyl ether, tetrahydrofuran and acetonitrile solutions. (e) Fluorescence and phosphorescence at 77 K. (f) Nanosecond-level lifetimes in non-doped powder and PMMA-doped films. The concentration of the diluted solutions was 1 × 10^−5^ M.

Details of the synthesis of DPXZ-PI and DPXZ-PICN are described in the SI. We measured the absorption and emission spectra of the DPXZ-PI and DPXZ-PICN molecules (Fig. 3a, S5 and S6). In contrast to the classic orthogonal-direction HLCT material TBPMCN, both DPXZ-PI and DPXZ-PICN exhibit two distinct absorption bands centered near 330 nm and 370 nm, respectively. Based on the characteristic absorption profiles of HLCT materials like TBPMCN, the band around 370 nm can be assigned to the HLCT absorption. The isolated peak at 330 nm also possesses a relatively high molar extinction coefficient. However, this feature cannot be attributed solely to a polycyclic π–π* transition, which would be located at around 280 nm. Considering the well-matched electronic properties of the strong DPXZ donor and the weak PI acceptor, the absorption near 330 nm is likely intrinsic to the DPXZ unit itself, consistent with the S_2_ NTO of DPXZ-PI. The absorption spectrum of DPXZ-PICN is broadly similar to that of DPXZ-PI, where the primary difference is the less resolved distinction between its two absorption bands, resulting in a single broad absorption peak. Solvatochromic emission spectra provide further critical insights into the nature of the HLCT state. For TPA-PPI and TBPMCN, the introduction or absence of the cyano group profoundly alters HLCT formation, dramatically affecting the peak shape, spectral width, and the magnitude of the solvatochromic shift. However, the co-axial HLCT materials DPXZ-PI and DPXZ-PICN exhibit very little difference aside from a *ca.* 5 nm disparity in the solvatochromic shift. Both DPXZ-PI and DPXZ-PICN display pronounced vibrational fine structure in non-polar hexane and significant spectral broadening in higher polarity solvents (Fig. 3b). The Lippert–Mataga model of the two materials further reveals comparable one-component lines corresponding to excited-state dipole moments of 18.8 debye and 19.9 debye for DPXZ-PI and DPXZ-PICN, respectively (Fig. 3c). This observation reinforces the concept of a robust co-axial HLCT character largely unaffected by peripheral cyano substitution. Furthermore, both materials exhibit single-exponential nanosecond-scale fluorescence decays in dilute solution (Fig. 3d), and their distinct phosphorescence emissions (Fig. 3e), resolvable from the fluorescence emissions, are detectable at the temperature of liquid nitrogen (77 K). The phosphorescence maxima occur at 542.6 nm for DPXZ-PI and 541.2 nm for DPXZ-PICN, corresponding to large singlet-triplet energy gaps (Δ*E*_st_) of 0.6392 eV and 0.6070 eV, respectively, which unequivocally demonstrate the typical HLCT characteristics of the materials.

A key advantage of the HLCT state lies in its elevated radiative decay rate constant (*k*_r_) and suppressed non-radiative decay rate constant (*k*_nr_) within moderate polarity environments, enabling high PLQYs in aggregates. This property constitutes the foundation for achieving high electroluminescence efficiency. As summarized in Table 2, both DPXZ-PI and DPXZ-PICN exhibit substantial *k*r values in moderate polarity solvents (ethyl ether and tetrahydrofuran (THF)) when compared to the value obtained in the low polarity solvent hexane, and thus the corresponding *k*_nr_ values are significantly suppressed. This behavior highlights the beneficial characteristics of the LE excited state properties, which effectively mitigate non-radiative decay even in relatively polar environments. This advantage is responsible for the hopeful result we obtained next where both DPXZ-PI and DPXZ-PICN achieved high PLQY values of 81.7% and 83.9%, respectively, when 1% of the DPXZ materials were doped in films of polymethyl methacrylate (PMMA). These values are approximately two-fold and 1.75-fold that of TBPMCN meaning that if the EUEs of OLEDs based on DPXZ-PI and DPXZ-PICN can be preserved as near to 100% as possible, the EQEs of the OLEDs can be the same magnitudes greater than an OLED based TBPMCN. Additionally, the introduction of the cyano group in DPXZ-PICN leads to a dramatic increase in the *k*_nr_ value only in the highly polar solvent acetonitrile, which indicates that the pronounced CT character of the co-axial HLCT could only be induced under extremely high polarity conditions, thus revealing it's the robustness of the material under different circumstances.

**Table 2:** Excited state dynamics of DPXZ-PI and DPXZ-PICN

Solvent	DPXZ-PI				DPXZ-PICN			
	PLQY (%)	T (ns)	*k*_r_ (10^8^ s^−1^)	*k*_nr_ (10^8^ s^−1^)	PLQY (%)	T (ns)	*k*_r_ (10^8^ s^−1^)	*k*_nr_ (10^8^ s^−1^)
Hexane	60.7	1.904	3.19	2.06	36.1	2.023	1.78	3.16
Ethyl ether	60.3	3.321	1.82	1.20	58.6	3.376	1.74	1.23
THF	53.4	3.482	1.53	1.34	60.4	3.740	16.1	1.06
Acetonitrile	23.5	4.208	0.60	1.82	4.3	0.810	0.50	11.8
PMMA	81.7	3.402	2.40	0.54	83.9	4.462	1.58	0.66
Neat film	100	4.826	0.65	1.42	30.2	0.923	0.61	10.2

### 2.3 OLED performance

Besides having a high PLQY, efficient charge carrier transport is also crucial for realizing higher efficiency OLEDs. Prior to OLED fabrication, we characterized the carrier mobility of DPXZ-PI and DPXZ-PICN by constructing and analyzing hole-only devices (HODs) and electron-only devices (EODs) using the space charge limited current (SCLC) method. On the one hand, due to the weak electron acceptor ability of the PI group, the average electron mobility (*μ*_ele_) of DPXZ-PI is estimated to be 1.86 × 10^−6^ cm^2^ V^−1^ s^−1^, which is a modest value for an organic emitter. DPXZ-PICN exhibited an average *μ*_ele_ value of 3.78 × 10^−6^ cm^2^ V^−1^ s^−1^ double that of DPXZ-PI, which can be assigned to the introduction of the electron-accepting cyano group. On the other hand, both of the materials demonstrate significantly high hole mobilities (*μ*_h_): DPXZ-PI and DPXZ-PICN achieved average *μ*_h_ values of 1.56 × 10^−4^ cm^2^ V^−1^ s^−1^ and 3.79 × 10^−4^ cm^2^ V^−1^ s^−1^, respectively. Therefore, despite their moderate electron mobilities, the excellent hole-transport capabilities of both materials suggest that high electroluminescence efficiency and low efficiency roll-off remain achievable through rational host matrix selection and optimized doping concentrations in device architectures. Electroluminescent (EL) materials must also possess excellent thermal stability to ensure they do not decompose (reflected by the thermal decomposition temperature, *T*_d_) or suffer from morphology degradation (reflected by the glass transition temperature, *T*_g_) during device fabrication. We performed thermogravimetric analysis (TGA) and differential scanning calorimetry (DSC) on both materials. Both compounds exhibited high *T*_d_ values of 460 °C and high *T*_g_ values of >120 °C, which are favorable for OLED fabrication (Table 3).

**Table 3:** Thermal stability parameters and energy levels of DPXZ-PI and DPXZ-PICN

	DPXZ-PI	DPXZ-PICN
*μ*_elec_ (cm^2^ V^−1^ s^−1^)	1.86 × 10^−6^	3.78 × 10^−6^
*μ*_hole_ (cm^2^ V^−1^ s^−1^)	1.56 × 10^−4^	3.79 × 10^−4^
*T*_g_ (°C)	>120 °C	>120 °C
*T*_d_ (°C)	460	458
HOMO (eV)	−5.08	−5.12
LUMO (eV)	−2.35	−2.30

**Fig. 4:**
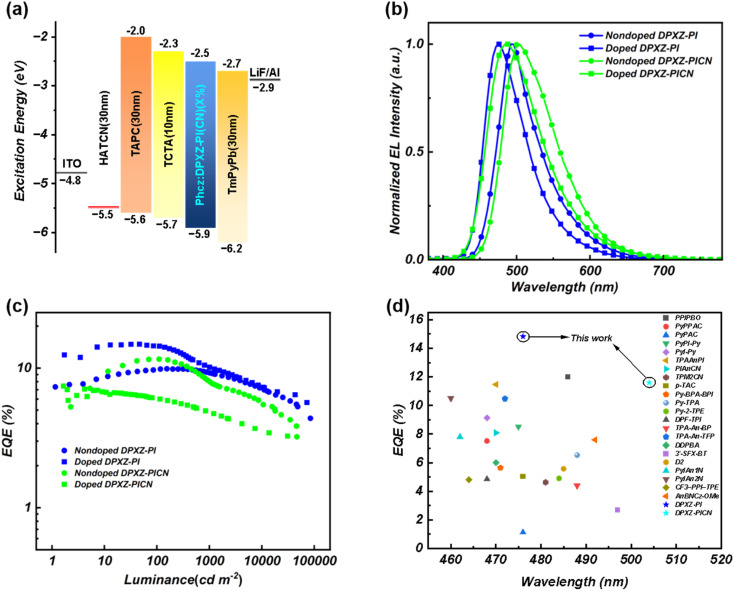
OLED performance of DPXZ-PI and DPXZ-PICN. (a) Schematic diagram of the OLED structure. (b) EL spectra. (c) EQE-EL relationship. (d) Comparison of the materials reported in this work with other reported HLCT material-based OLEDs.^68–86^

Accordingly, we initially fabricated non-doped OLEDs based on DPXZ-PI and DPXZ-PICN with the following structures: ITO/HATCN (5 nm)/TAPC (25 nm)/TCTA (15 nm)/DPXZ-PI (20 nm)/TmPyPb (35 nm)/LiF (1 nm)/Al (100 nm) and ITO/HATCN (5 nm)/TAPC (30 nm)/TCTA (15 nm)/DPXZ-PICN (20 nm)/TmPyPb (30 nm)/LiF (1 nm)/Al (100 nm). As shown in Fig. 4 and Table 4, The DPXZ-PI-based non-doped device achieved a maximum EQE of 9.9% with Commission Internationale de l’Eclairage (CIE) coordinates of (0.207, 0.459). In an attempt to obtain high performance HLCT material-based OLEDs, we further proceeded to fabricate doped OLEDs utilizing PhCzBCz as the host matrix. The following device structures were used: ITO/HATCN (5 nm)/TAPC (25 nm)/TCTA (10 nm)/PhCzBCz: DPXZ-PI/TmPyPb (35 nm)/LiF (1 nm)/Al (100 nm) and ITO/HATCN (5 nm)/TAPC (30 nm)/TCTA (10 nm)/PhCzBCz: DPXZ-PICN/TmPyPb (30 nm)/LiF (1 nm)/Al (100 nm). The DPXZ-PI-doped OLED delivered a remarkable maximum EQE of 14.8%, with CIE coordinates of (0.166, 0.280). This performance represents the highest reported EQE for sky blue-emitting OLEDs with a comparable molecular structure to date. For DPXZ-PICN, notably, despite the sharp quenching observed in the non-doped powder, the DPXZ-PICN-based non-doped OLED can also reach a record-setting maximum EQE of 11.67% with a blue-greenish EL. Meanwhile, the doped OLED based on DPXZ-PICN exhibited a significant performance reduction (maximum EQE of only 7.4%) (Fig. 4, and Table 4). These seemingly reversed EQE results are complicated and are probably related to the electric-field regulation effect,^67^ but in terms of the HLCT excited state, cyano-substitution of DPXZ-PI does not have much effect on the EQE, again revealing the robustness of the co-axial HLCT construction method (Table 5).

**Table 4:** DPXZ-PI- and DPXZ-PICN-based OLED performance

Device	*V*_on_ (V)	LE_max_^e^ (cd A^−1^)	PE_max_^f^ (lm W^−1^)	CIE (*x*, *y*)	*L*_max_ g (cd m^−2^)	EQE_max_ (%)	PLQY (%)	Roll-off^h^ (%)
1^a^	2.8	25.59	22.3	(0.207, 0.459)	84 225	9.9	100	N. A./6.3
2^b^	3.2	29.22	25.90	(0.166, 0.280)	72 406	14.8	81.7	2.6/31.8
3^c^	3	34.19	30.69	(0.256, 0.535)	46 519	11.6	30.2	N. A./31.4
4^d^	3.8	17.99	14.87	(0.203, 0.376)	31 998	7.4	83.9	18.4/34.8

a Non-doped OLED based on DPXZ-PI.

b Doped OLED based on DPXZ-PI.

c Non-doped OLED based on DPXZ-PICN.

d Doped OLED based on DPXZ-PICN.

e maximum luminescent efficiency.

f Maximum powder efficiency.

g Maximum luminescence.

h Efficiency roll-off recorded at luminescences of 100 cd m^−2^ chem and 1000 cd m^−2^, N. A. means no efficiency roll-off or minus efficiency roll-off is recorded at 100 cd m^−2^.

**Table 5:** Summary of the lifetimes of DPXZ-PI and DPXZ-PICN

Temperature (K)	DPXZ-PI					DPXZ-PICN				
	*τ*_p_ (ns)	*τ*_d_ (ms)				*τ*_p_ (ns)	*τ*_d_ (ms)			
	τ (ns)	*τ*_1_ [ms]/Rel%	*τ*_2_ [ms]/Rel%	*τ*_3_ [ms]/Rel%	*τ*_aver_ [ms]	*τ* (ns)	*τ*_1_ [ms]/Rel%	*τ*_2_ [ms]/Rel%	*τ*_3_ [ms]/Rel%	*τ*_aver_ [ms]
80	3.895	0.211/43.82%	1.924/56.18%	—	1.173	4.736	0.659/58.72%	5.858/41.28%	—	2.774
150	3.919	0.059/15.05%	0.482/49.33%	3.325/35.62%	1.431	4.758	0.332/30.52%	1.730/41.26%	18.91/28.23%	6.153
220	3.995	0.076/17.93%	0.558/49.19%	3.817/32.88%	1.543	4.814	0.325/29.09%	1.822/40.07%	22.37/30.83%	7.722
290	3.997	0.111/24.23%	0.753/48.02%	5.427/27.75%	1.894	4.833	0.391/31.65%	2.148/36.86%	20.45/31.49%	7.355

### 2.4 mechanism of exciton utilization in the OLEDs

**Fig. 5:**
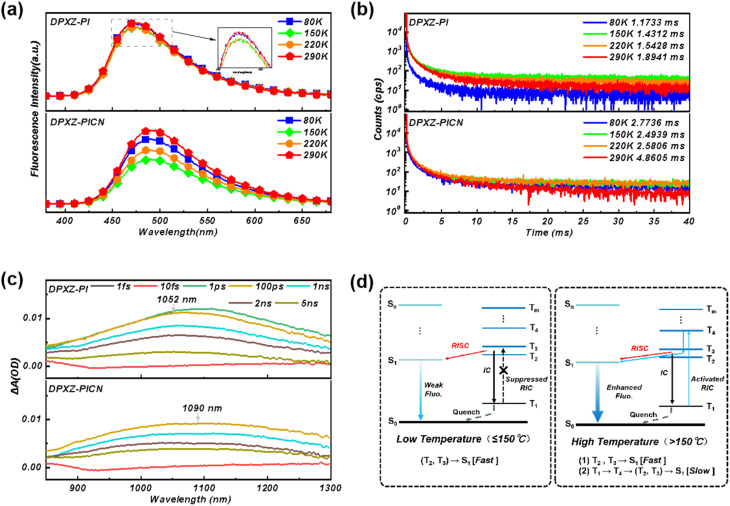
(a) Temperature-dependent PL spectra of DPXZ-PI and DPXZ-PICN. (b) Temperature-dependent lifetimes of DPXZ-PI and DPXZ-PICN within a time window of 40 ms. (c) Ultrafast absorption of DPXZ-PI and DPXZ-PICN with Ir (ppy)_3_ as the sensitizer. (d) Schematic diagrams of the hRISC channel in DPXZ-PI and DPXZ-PICN. The concentration of the diluted solutions was 1 × 10^−5^ M.

Considering the high EQE of the non-doped OLED based on DPXZ-PI and the doped OLED based on DPXZ-PICN, the EUEs of their respective OLEDs would no doubt exceeded the spin-statistics limit of 25%. This finding can be assigned to their hRISC pathway from the LE state *T*_4_ to the HLCT state S_1_ that originates from the DPXZ-PI backbone.^87^ To further understand the “hot-exciton” channels in DPXZ-PI and DPXZ-PICN, we conducted temperature-dependent photoluminescence (PL) spectroscopy and PL lifetime measurements on both materials. First, the PL intensity of both DPXZ-PI and DPXZ-PICN initially decreased and subsequently increased with rising temperature (Fig. 5a).^88^ The initial PL decrease before 150 °C can be assigned to activated molecular motion upon temperature increase, and the subsequent PL increase originates from a temperature-activated RISC process. This mechanism is further corroborated by the temperature-dependent lifetime results. In Fig. 5b, significant millisecond lifetime species can be observed in a 40 ms time window, and extra lifetime species can further be observed at temperatures >80 K. These findings are consistent with our previous work on D–π–A HLCT materials^43^ that showed that the extra lifetime species are critically linked to the thermally activated reversed interconversion (RIC) process. To examine the RIC pathway, we carried out ultrafast absorption spectroscopy using Ir(ppy)_3_-sensitized solutions of DPXZ-PI and DPXZ-PICN (Fig. S17). As shown in Fig. 5c, for both the DPXZ-PI- and DPXZ-PICN-sensitized solutions, broad stationary absorption bands that remain invariant over time can be observed in the near-infrared (NIR) region, corresponding to *T*_1_ → *T*_*n*_ transition energies of 1.1787 eV and 1.1376 eV, respectively. By referencing the energy level diagrams in Fig. 1a, the *T*_1_ → *T*_*n*_ transitions revealed by the ultrafast absorptions of DPXZ-PI and DPXZ-PICN can be assigned to their *T*_1_ → *T*_4_ transitions, which also comply with the El-Sayed rule judging from the similar π → π* transition pattern of *T*_1_ and *T*_4_ (Fig. S2).^89–92^

## 3 Conclusion

In summary, we have reported the synthesis and theoretical and experimental investigation of two highly efficient electro-fluorescent materials DPXZ-PI and DPXZ-PICN with excited state designs based on co-axial HLCT. The matching of a strong donor and weak acceptor helps create more orbital overlap and a higher PLQY while maintaining the CT character required for a high EUE. This final property in particular is the key reason for the high EQE of 14.8% for the doped OLED based on DPXZ-PI. In addition, the co-axial HLCT state remains stable, even upon substitution of the strong acceptor with a cyano group, and this enables the non-doped OLED based on DPXZ-PICN to obtain an EQE of 11.6%. Due to the advantages of the high PLQY and EUE, the co-axial HLCT mechanism is expected to be a novel method for the design of cost-effective and easily scalable next-generation electro-fluorescent materials.

## Conflicts of interest

There are no conflict to declare.

## Supplementary Material

SC-OLF-D5SC06557G-s001

SC-OLF-D5SC06557G-s002

## Data Availability

All relevant data are either included in the main text or in the supporting information (SI). Supplementary information is available. See DOI: https://doi.org/10.1039/d5sc06557g.
