# Supplementary figures and images for: Realizing highly efficient electrofluorescence through a co-axial hybrid local and charge-transfer (HLCT) excited state
Source: Chem Sci. 2025 Oct 10;16(47):22679–89. doi: 10.1039/d5sc06557g (PMC12570977; doi:10.1039/d5sc06557g)

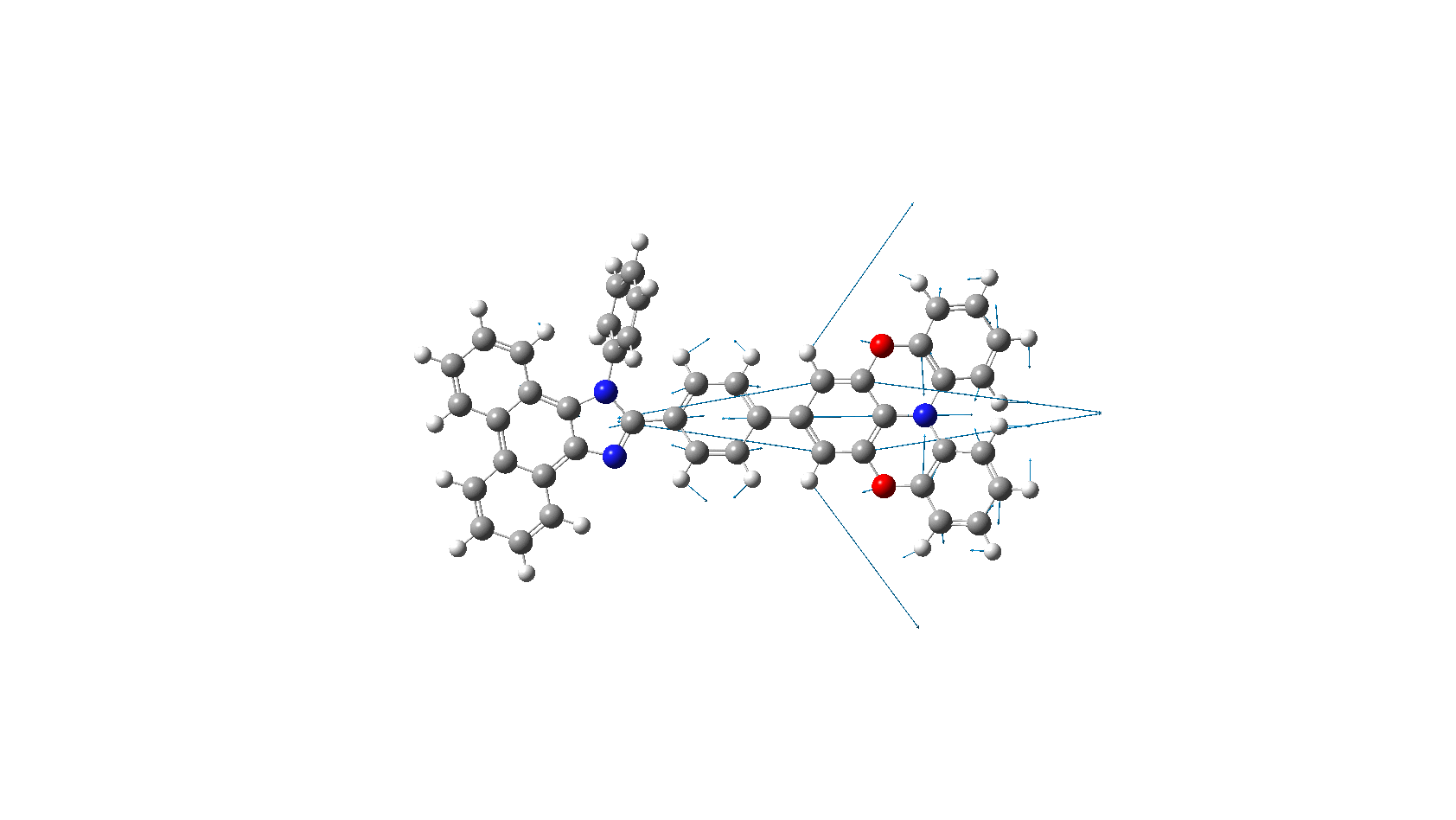

Supplement: SC-016-D5SC06557G-s001 [file SC-016-D5SC06557G-s001.zip › Animation of vibration modes for DPXZ-PI, DPXZ-PICN, TPA-PPI, and TBPMCN/DPXZ-PI/1752.95cm-1.gif]

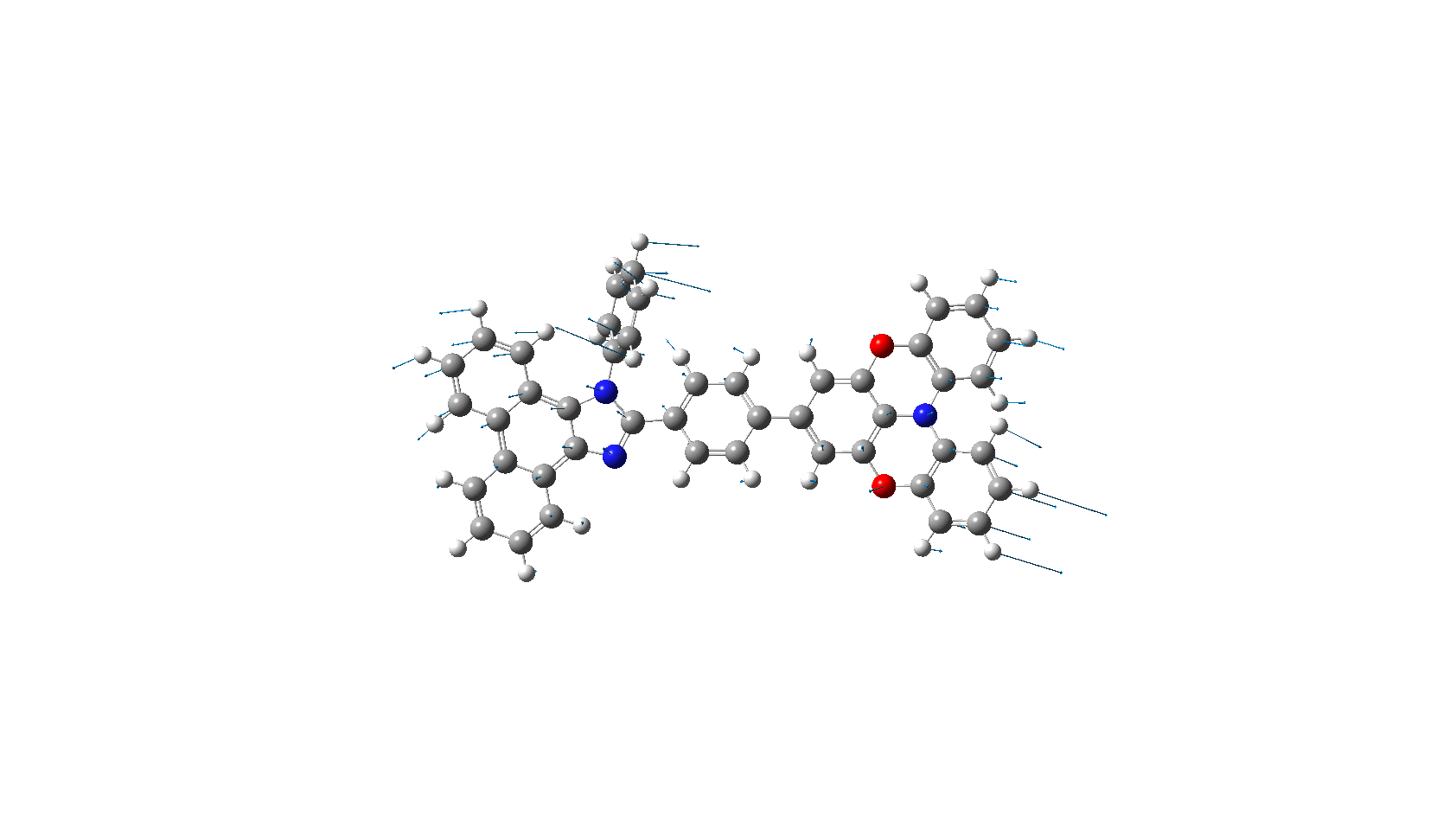

Supplement: SC-016-D5SC06557G-s001 [file SC-016-D5SC06557G-s001.zip › Animation of vibration modes for DPXZ-PI, DPXZ-PICN, TPA-PPI, and TBPMCN/DPXZ-PI/79.16cm-1.gif]

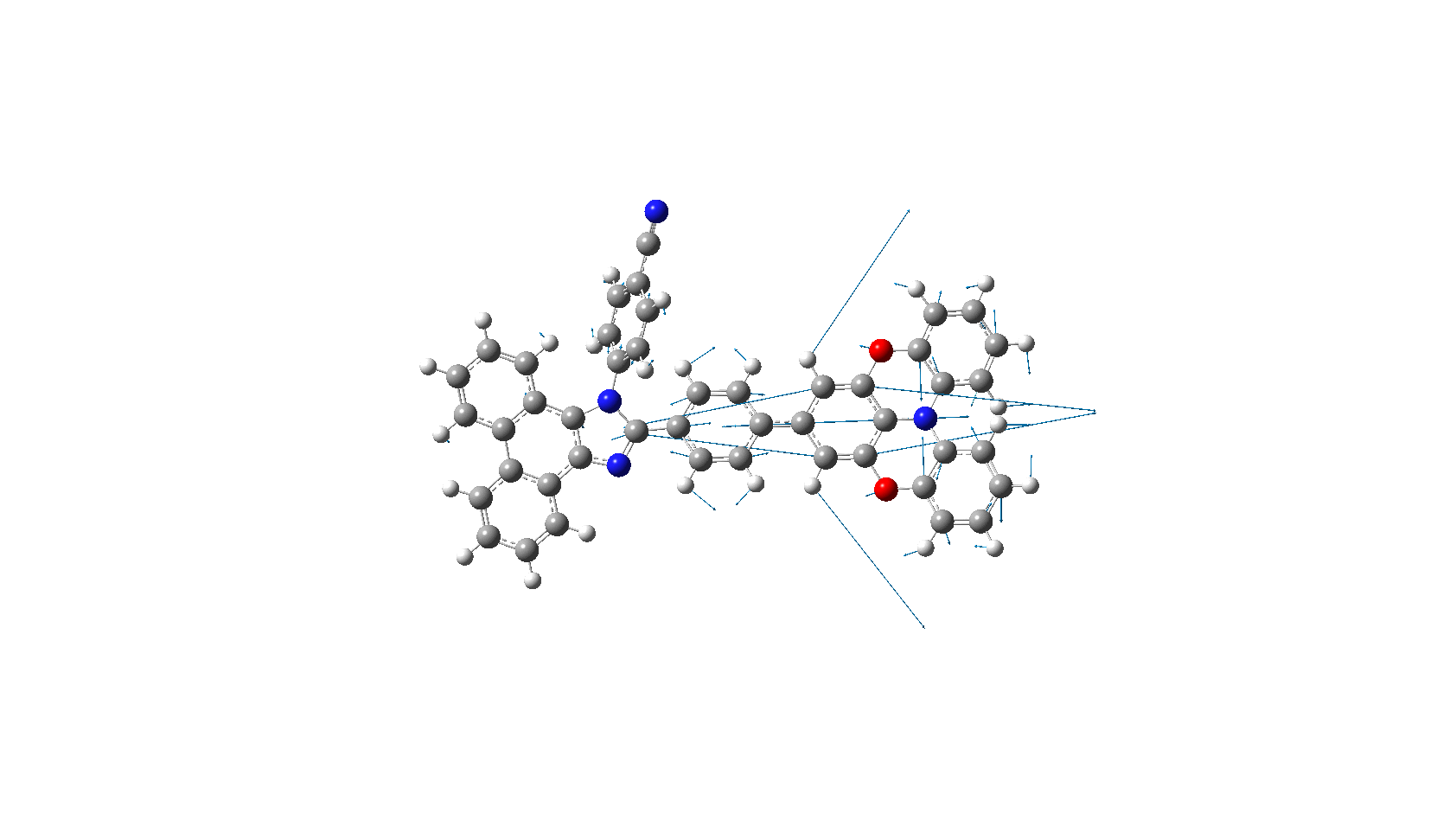

Supplement: SC-016-D5SC06557G-s001 [file SC-016-D5SC06557G-s001.zip › Animation of vibration modes for DPXZ-PI, DPXZ-PICN, TPA-PPI, and TBPMCN/DPXZ-PICN/1751.03cm-1.gif]

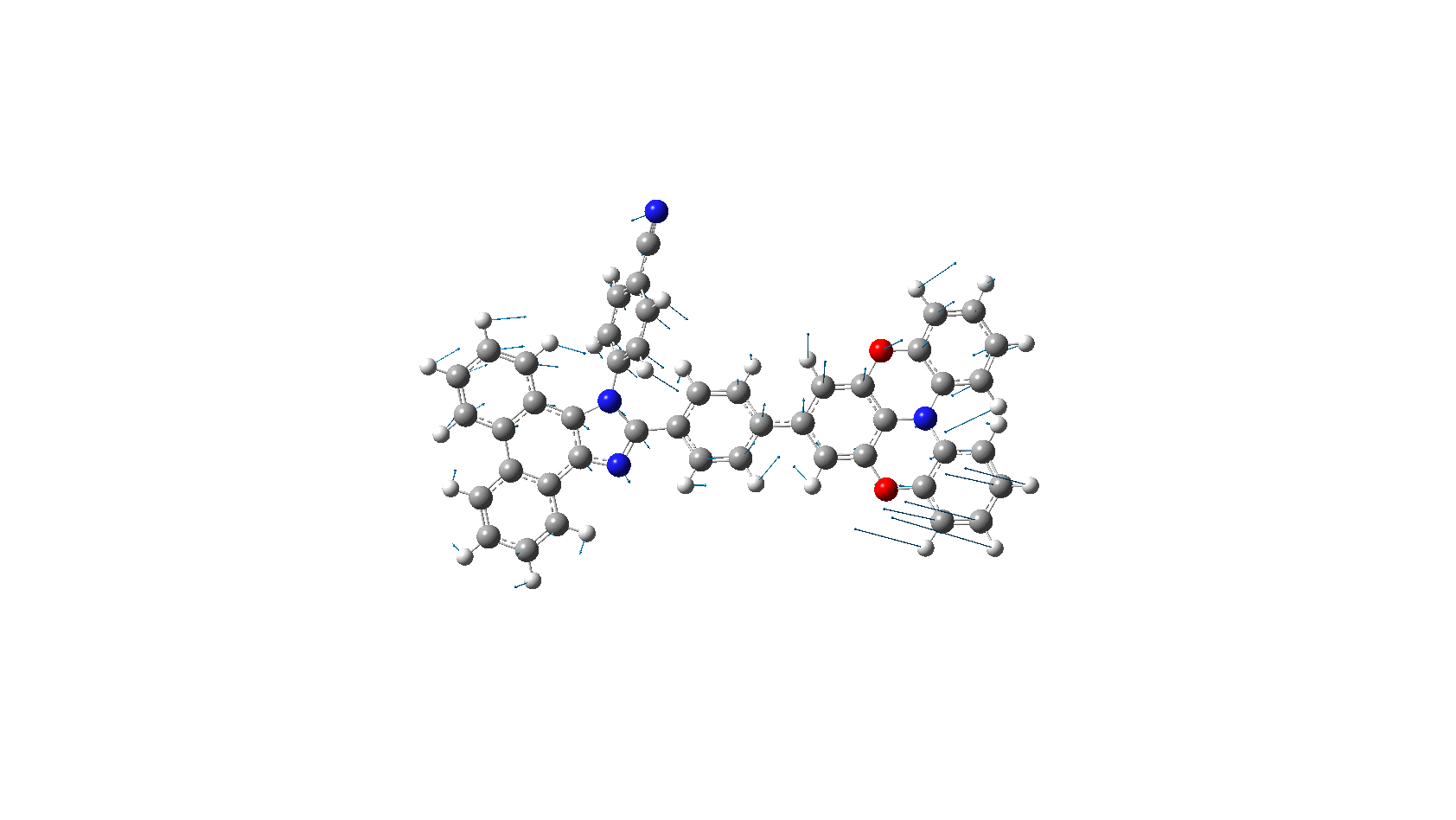

Supplement: SC-016-D5SC06557G-s001 [file SC-016-D5SC06557G-s001.zip › Animation of vibration modes for DPXZ-PI, DPXZ-PICN, TPA-PPI, and TBPMCN/DPXZ-PICN/78.91cm-1.gif]

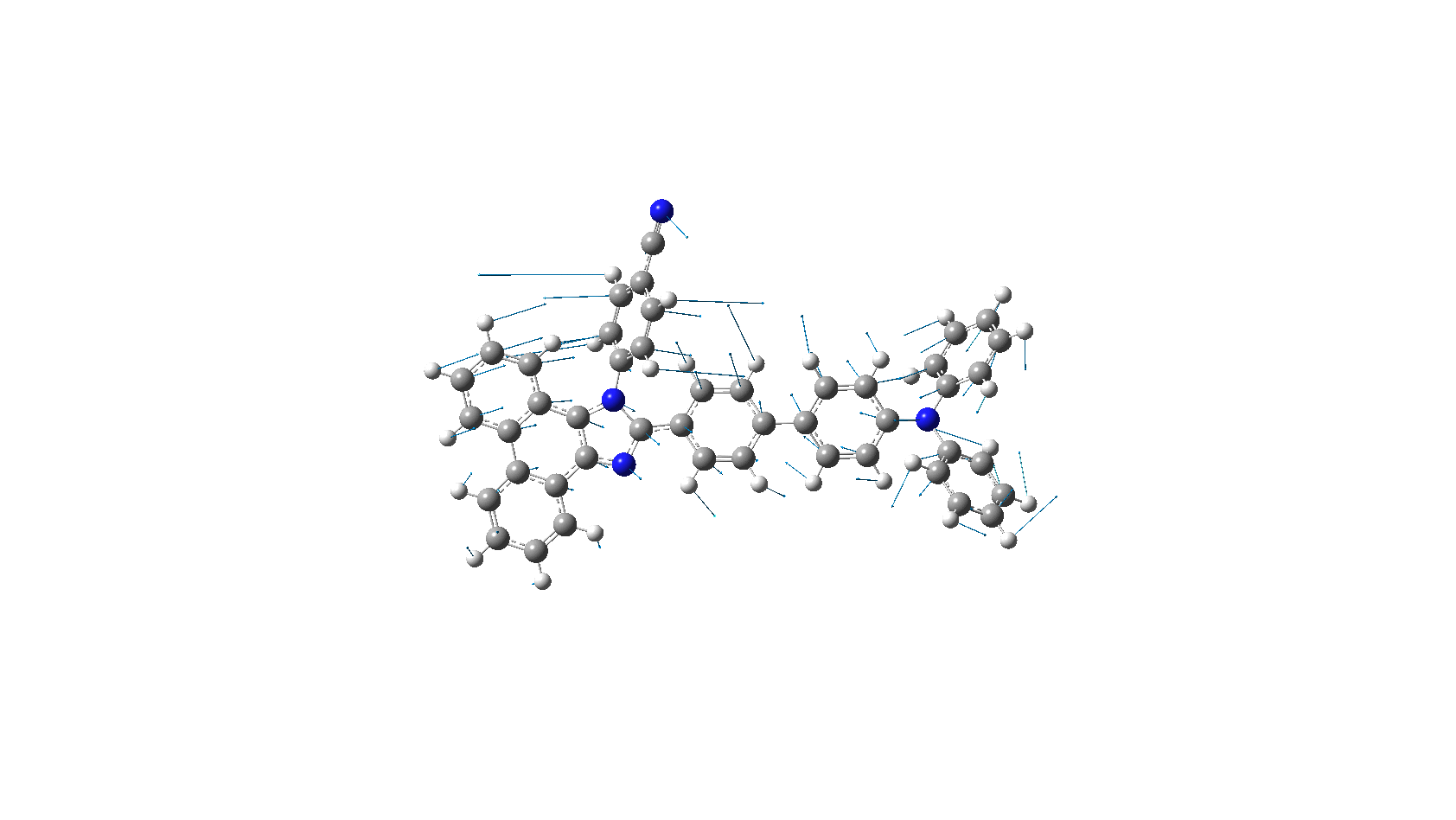

Supplement: SC-016-D5SC06557G-s001 [file SC-016-D5SC06557G-s001.zip › Animation of vibration modes for DPXZ-PI, DPXZ-PICN, TPA-PPI, and TBPMCN/TBPMCN/151.95cm-1.gif]

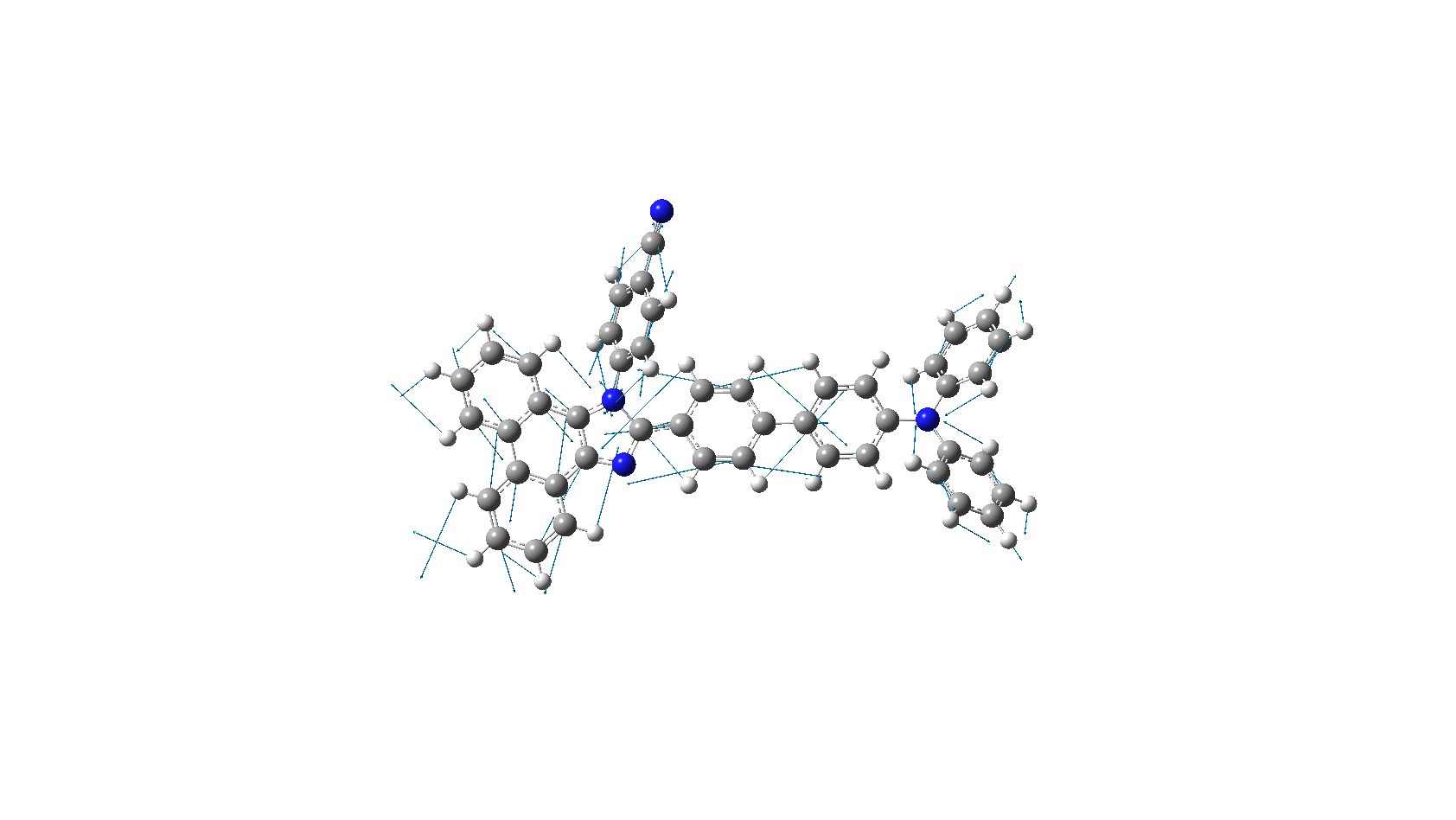

Supplement: SC-016-D5SC06557G-s001 [file SC-016-D5SC06557G-s001.zip › Animation of vibration modes for DPXZ-PI, DPXZ-PICN, TPA-PPI, and TBPMCN/TBPMCN/1698.79cm-1.gif]

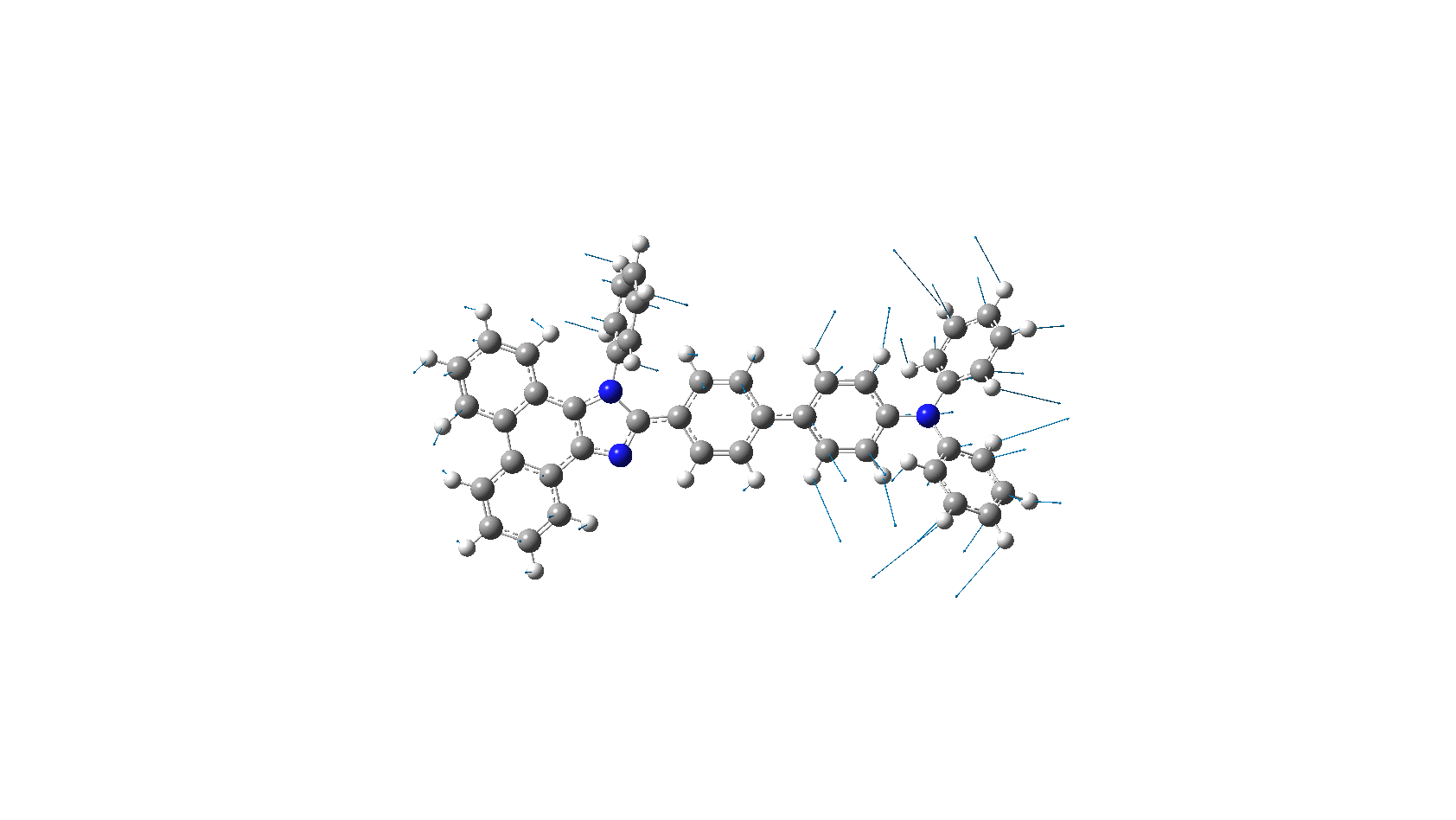

Supplement: SC-016-D5SC06557G-s001 [file SC-016-D5SC06557G-s001.zip › Animation of vibration modes for DPXZ-PI, DPXZ-PICN, TPA-PPI, and TBPMCN/TPA-PPI/107.87cm-1.gif]

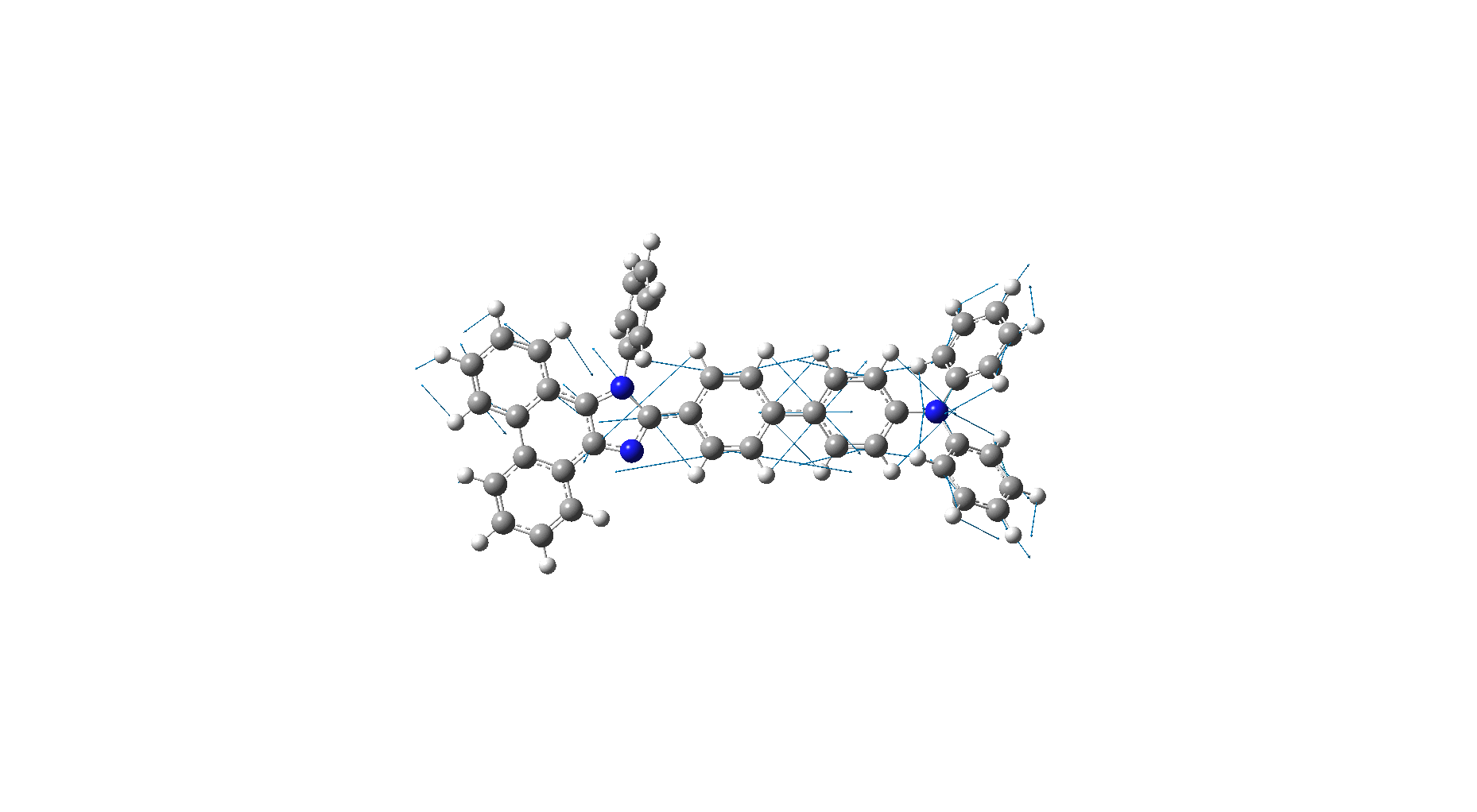

Supplement: SC-016-D5SC06557G-s001 [file SC-016-D5SC06557G-s001.zip › Animation of vibration modes for DPXZ-PI, DPXZ-PICN, TPA-PPI, and TBPMCN/TPA-PPI/1702.75cm-1.gif]
