# Supplementary material for: Realizing highly efficient electrofluorescence through a co-axial hybrid local and charge-transfer (HLCT) excited state
Source: Chem Sci. 2025 Oct 10;16(47):22679–89. doi: 10.1039/d5sc06557g (PMC12570977; doi:10.1039/d5sc06557g)
Supplement: SC-016-D5SC06557G-s002 [file SC-016-D5SC06557G-s002.pdf]

Supplementary Information (SI) for Chemical Science.
This journal is © The Royal Society of Chemistry 2025

Supporting Information

Realizing Highly Efficient Electro-Fluorescence with Co-axial Hybrid Local and Charge-transfer (HLCT) Excited State

Kuo Yu [1], Yingbo Lv [1], Yilong Li [1], Zirui Wang [1], Shi-Tong Zhang [1,*], Jinbei Wei [2,*], Shanfeng Xue [3,*], Chenguang Wang[2] and Bing Yang[1]

1 *State Key Laboratory of Supramolecular Structure and Material, College of Chemistry, Jilin University, Changchun, 130012, P. R. China*

2 *State Key Laboratory of Integrated Optoelectronics, JLU Region, College of Electronic Science and Engineering, Jilin University, Changchun, 130012, China*

3 *Key Laboratory of Rubber-Plastics of the Ministry of Education, School of Polymer Science & Engineering, Qingdao University of Science and Technology, 53 Zhengzhou Road, Qingdao 266042, P. R. China*

Email: stzhang@jlu.edu.cn, jinbwei@jlu.edu.cn, sfxue@qust.edu.cn

1. Experimental Procedures

General Information

All the reagents and solvents used for the synthesis were purchased from energy-chemical and used without further purification. The nuclear magnetic resonance (NMR) spectra were recorded on a Bruker AVANCE 500 spectrometer using DMSO-d_6 as solvent. The high-resolution mass spectra (HRMS) were recorded using an ITQ1100 (Thermo Fisher).

Cyclic voltammetry

Cyclic voltammetry (CV) was performed with a BAS 100W Bioanalytical Systems in a solution of tetra-n-butylammonium-hexafluorophosphate (Bu_4NPF_6) (0.1 M) in anhydrous dimethylformamide (DMF) or anhydrous dichloromethane (DCM) at a scan rate of 100mV s^{-1}, using a platinum wire auxiliary electrode, a glass carbon disk as the working electrode, and Ag/Ag^+ as the reference electrode. The HOMO/LUMO levels were calculated according to the following formalism:

$$E_{HOMO}/eV = -(E_{ox} \text{ vs. } Fc/Fc^+ + 4.8)$$

$$E_{LUMO}/eV = -(E_{red} \text{ vs. } Fc/Fc^- + 4.8)$$

where E_{HOMO} and E_{HOMO} are the HOMO and LUMO energy; E_{ox} and E_{red} are the oxidation offset and reduction onset, whose units are V; $E(Fc/Fc^+)$ and $E(Fc/Fc^-)$ are the half-wave potential of ferrocene, whose units are also V.

Photoluminescence quantum yield (PLQY)

Photoluminescence quantum yield (PLQY) for thin film was measured using an integrating sphere apparatus. PLQY of different solutions were determined by using 0.1 M quinine sulfate as a reference (PLQY= 54.6%) and were calculated by using the following formula:

$$Q_x = Q_r\left(\frac{A_r(\lambda_r)}{A_x(\lambda_x)}\right)\left(\frac{I(\lambda_r)}{I(\lambda_x)}\right)\left(\frac{n_x^2}{n_r^2}\right)\left(\frac{D_x}{D_r}\right)$$

Where Q is the PLQY, A is the value of absorbance, I is the intensity of excitation source, n is the refractive index of solvent, D is the area of emission spectra, λ is the corresponding wavelength. The subscript *r* stands for the reference while x stands for test subject. The excitation wavelength is 365 nm. Radiative transition rate (k_r) and non-radiative transition rate (k_{nr}) were calculated according to the following formula:

$$k_r = \frac{\Phi}{\tau}$$

$$k_{nr} = \frac{1 - \Phi}{\tau}$$

(Φ: PLQY, τ: fluorescent lifetime).

Additionally, due to that oxygen quenching cannot be neglected for the neat films and the doped films of DPXZ-PI and DPXZ-PICN (Figure S19), we corrected the PLQY results measured in air according to the following formula:

$$PLQY_{vac} = \frac{A_{vac}}{A_{air}} PLQY_{air}$$

Where $PLQY_{vac}$ is the corrected PLQY under vacuum, A_{air} is the Emission peak area in air, A_{vac} is the Emission peak area under vacuum, and the $PLQY_{air}$ is the PLQY measured in air.

Lippert-Mataga solvatochromic model

The influence of solvent polarity on the photophysical properties of DPXZ-PI(CN) was analyzed by the Lippert-Mataga equation, which can describe the interactions between the solvent and the dipole moment of solute.

$$hc(\nu_a - \nu_f) = hc(\nu_a^0 - \nu_f^0) - \frac{2(\mu_e - \mu_g)^2}{a^3} f(\varepsilon, n)$$

where f is the orientational polarizability of the solvent; μ_e is the excited-state dipole moment; μ_g is the ground state dipole moment; a is the solvent cavity (Onsager) radius, derived from the Avogadro number (N), molecular weight (M), and density (d = 1.0 g/cm^3); ε and n are the solvent dielectric and the solvent refractive index,

respectively; and f (ε, n) and a can be calculated, respectively, as the following:

$$f(\varepsilon, n) = \frac{\varepsilon - 1}{2\varepsilon + 1} - \frac{n^2 - 1}{2n^2 + 1}$$

$$a = \left(\frac{3M}{4N\pi d}\right)^{\frac{1}{3}}$$

Spectroscopy measurements

UV–vis spectra of solutions were recorded on a Shimadzu UV-3100 Spectrophotometer. Steady-state photoluminescence (PL) spectra, time-resolved PL spectra and temperature-dependent PL spectra were carried out by FLS980 Fluorescence Spectrometer by Edinburgh Instruments.

Theoretical calculation

The geometry optimization and excites state estimation are carried out using a Gaussian 16 A.03 package[1] under m062x/6-31 g (d, p) and td-m062x/6-31g (d, p) level, respectively. The natural transition orbital (NTO) images are generated using a MultiWFN 3.8 dev package[2].

Calculation on the Spin-orbit Coupling (SOC) coefficient

The spin-orbit coupling (SOC) coefficient is calculated using a Beijing Density Function (BDF) package under td-m062x/6-31g (d, p) level[3]. For multi-electron atoms, a commonly used approximation is LS coupling (also known as Russell-Saunders coupling). The total SOC Hamiltonian is the sum of the SOC contributions from all electrons:

$$\hat{H}_{SOC} \approx \sum_i \zeta(\gamma_i)\hat{l}_i \cdot \hat{s}_i$$

In some cases, it can be approximated:

$$\hat{H}_{SOC} \approx \lambda \hat{L} \cdot \hat{S}$$

where $\hat{L}$ and $\hat{S}$ represent the total orbital angular momentum and total spin angular momentum, respectively, and λ is the effective SOC constant.

Estimation of LE and CT component

The LE and CT components of TBPMCN, DPXZ-PI and DPXZ-PICN are estimated

using the inter-fragment charge transfer (IFCT) method basing on the MultiWFN 3.8 dev package. To reveal the difference of the HLCT formation between the co-axial and orthogonal CT directions, we adopt two different fragment division methods, which are depicted as follows:

Fragment definition for calculation of the CT proportion

Molecule	Structure	"Co-axial" Donor	"Co-axial" Acceptor	"Orthogonal" Donor	"Orthogonal" Acceptor
TBPMCN	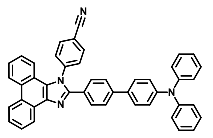	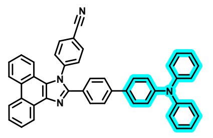	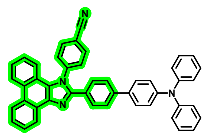	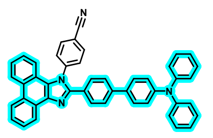	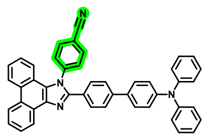
DPXZ-PI	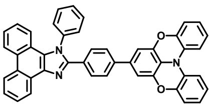	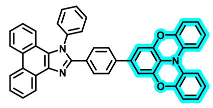	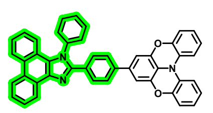	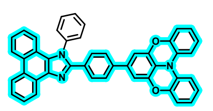	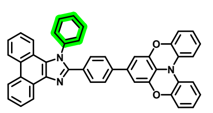
DPXZ-PICN	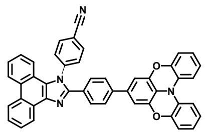	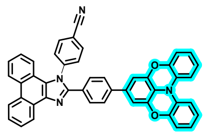	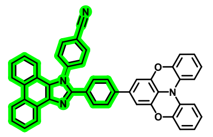	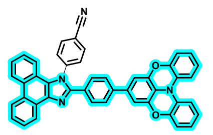	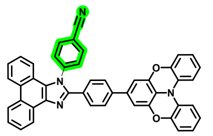

Determination of the Space-Charge Limited Current (SCLC) region of a single-carrier device

The SCLC region in unipolar devices can be identified by analyzing the J-V curve. For ideal, trap-free unipolar devices (such as electron-only or hole-only devices) under ohmic contacts and space-charge-dominated conditions, the current density (J) and applied voltage (V) satisfy:

$$J = (\frac{9}{8}) \times \varepsilon \times \mu \times (\frac{V^2}{L^3})$$

Which indicates that at a fixed device thickness (L), current density is proportional to the square of the voltage. When measuring the current density-voltage (J-V) characteristics of a unipolar device at a fixed thickness (L) and plotting a double-logarithmic graph with log(J) as the vertical axis and log(V) as the horizontal axis, if a distinct linear region appears where the slope (n) approaches 2 ($\log(J) \propto 2 * \log(V)$ or $J \propto V^2$), this voltage range corresponds to the

Space-Charge Limited Current (SCLC) region.

Synthesis and structural characterization

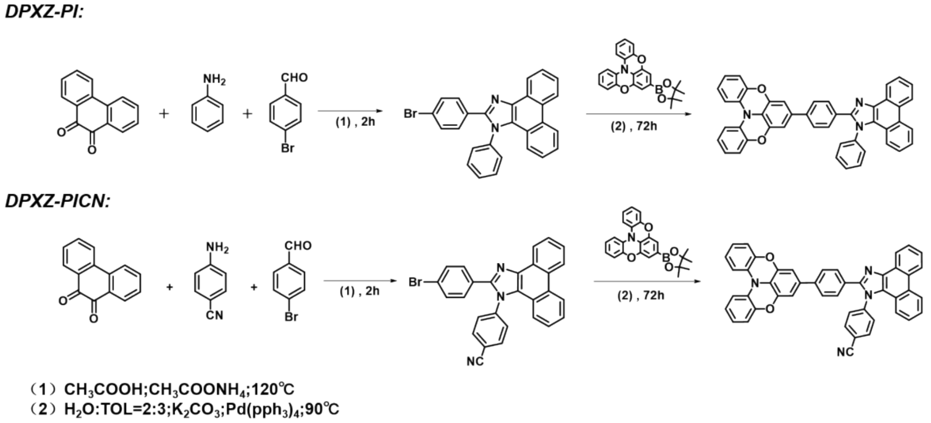

Scheme S1. Synthetic routes for target products and intermediate products.

Synthesis of 4-(2-(4-Bromophenyl)-1H-Phenanthro[9,10-d]Imidazol-1-yl)Benzene(BrBPI) :

A mixture of 10.0 mmol (1.84g) 4-bromobenzaldehyde, 10.0 mmol (2.08 g) phenanthrenequinone, 40 mmol (3.72g) 4-aminobenzene, and 50 mmol (3.70 g) ammonium acetate with 40 mL acetic acid were added into a clean 250 mL flask and refluxed under N2 in a 120 °C oil bath for 2 h. After cooling down, the solid product was filtrated and washed with 60 mL 1:1 water/acetic acid and 60 mL water successively, dissolved in CH2Cl2 and dried in MgSO4 overnight, purified by thin layer chromatography and 3.82 g (8.5 mmol) white product was obtained, yield 85%: MS: 474.1287 (M(H +)), 1H NMR (500 MHz, DMSO-d6) δ 8.94 (d, J = 8.2 Hz, 1H), 8.89 (d, J = 8.2 Hz, 1H), 8.69 (dd, J = 7.9, 1.4 Hz, 1H), 7.79 (ddd, J = 8.0, 6.9, 1.0 Hz, 1H), 7.77 – 7.67 (m, 6H), 7.62 – 7.55 (m, 3H), 7.55 – 7.49 (m, 2H), 7.35 (ddd, J = 8.2, 6.9, 1.2 Hz, 1H), 7.09 (dd, J = 8.4, 1.3 Hz, 1H).

Synthesis of 4-(2-(4-Bromophenyl)-1H-Phenanthro[9,10-d]Imidazol-1-yl)Benzonitrile (BrBPMCN) :

The synthetic method of the compound was consistent with that described in Zhang et al.'s paper. A mixture of 10.0 mmol (1.84g) 4-bromobenzaldehyde, 10.0 mmol (2.08 g)

phenanthrenequinone, 40 mmol (4.72g) 4-aminobenzonitrile, and 50 mmol (3.70 g) ammonium acetate with 40 mL acetic acid were added into a clean 250 mL flask and refluxed under N2 in a 120 °C oil bath for 2 h. After cooling down, the solid product was filtrated and washed with 60 mL 1:1 water/acetic acid and 60 mL water successively, dissolved in CH2Cl2 and dried in MgSO4 overnight, purified by thin layer chromatography and 4.26 g (9 mmol) white product was obtained, yield 90%，The crude product was subjected to the next step without further purification.

Synthesis of DPXZ-PI :

A mixture of 1.5 mmol (638 mg) BrBPI, 1.5 mmol (600 mg) 7-(4,4,5,5-tetramethyl-1,3,2-dioxaborolan-2-yl)benzo[5,6][1,4]oxazino[2,3,4-kl]phenoxazine, 25 mmol (3.45 g) potassium carbonate, and 70 mg (0.06 mmol) tetrakis-(triphenylphosphine)-palladium(0) (Pd(PPh3)4) with 10 mL deionized water and 15 mL toluene were added into a clean 100 mL flask and refluxed for three days under N2 atmosphere. The organic phase was washed with 20 mL water and extracted with CH2Cl2 , then was dried in MgSO4 overnight and purified by thin layer chromatography. Finally, 720 mg (1.13 mmol) pure dry yellow-green product was obtained, yield75%. MS: 642.2302 (M(H +)) 1H NMR (500 MHz, DMSO-d6) δ 8.95 (d, J = 8.5 Hz, 1H), 8.89 (d, J = 8.4 Hz, 1H), 8.73 (d, J = 7.9 Hz, 1H), 7.82 – 7.62 (m, 10H), 7.57 (t, J = 7.6 Hz, 1H), 7.41 (d, J = 8.0 Hz, 2H), 7.35 (t, J = 7.7 Hz, 1H), 7.10 – 6.99 (m, 8H), 5.77 (d, J = 1.5 Hz, 1H).

Synthesis of DPXZ-PICN :

A mixture of 1.5 mmol (711 mg) BrBPMCN, 1.5 mmol (600 mg) 7-(4,4,5,5-tetramethyl-1,3,2-dioxaborolan-2-yl)benzo[5,6][1,4]oxazino[2,3,4-kl]phenoxazine, 25 mmol (3.45 g) potassium carbonate, and 70 mg (0.06 mmol) tetrakis-(triphenylphosphine)-palladium(0) (Pd(PPh3)4) with 10 mL deionized water and 15 mL toluene were added into a clean 100 mL flask and refluxed for three days under N2 atmosphere. The organic phase was washed with 20 mL water and extracted with CH2Cl2 , then was dried in MgSO4 overnight and purified by thin layer chromatography. Finally,750 mg (1.12 mmol) pure dry yellow-green product was obtained, yield75%. MS: 666.0755(M(H +)) 1H NMR (500 MHz, DMSO-d6) δ 8.97 (d, J = 8.5 Hz, 1H), 8.91 (d, J = 8.4 Hz, 1H), 8.72 (d, J = 8.2 Hz, 1H), 8.23 (d, J = 8.2 Hz, 2H), 8.05 (d, J = 8.1 Hz, 2H), 7.80 (t, J = 7.4 Hz, 1H), 7.71 (d, J = 8.1 Hz, 3H), 7.59 (t, J = 8.2 Hz, 3H),

7.42 (d, J = 7.8 Hz, 3H), 7.12 – 6.99 (m, 9H).

2. Figures and Tables

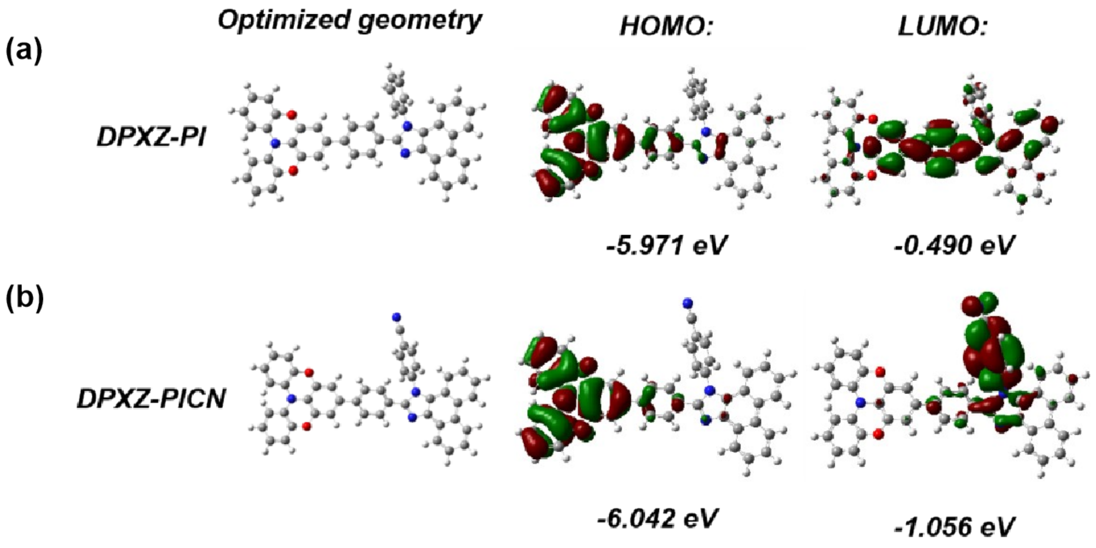

Figure S1. The optimized geometries and frontier molecular orbitals (FMO) of (a) DPXZ-PI and (b) DPXZ-PICN with calculated HOMO/LUMO distributions and corresponding energy.

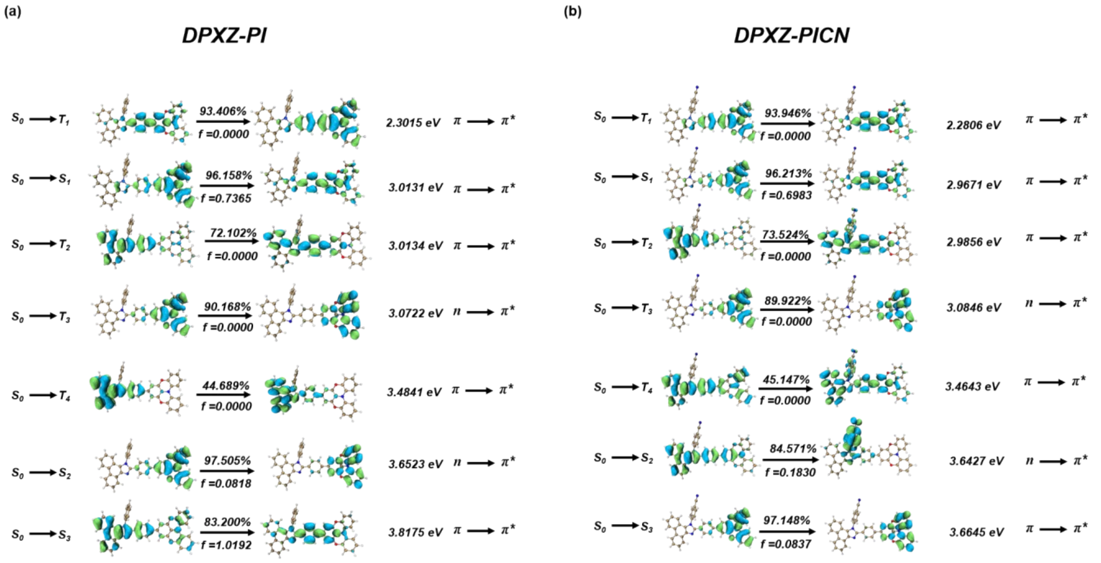

Figure S2. The NTO of (a) DPXZ-PI and (b) DPXZ-PICN.

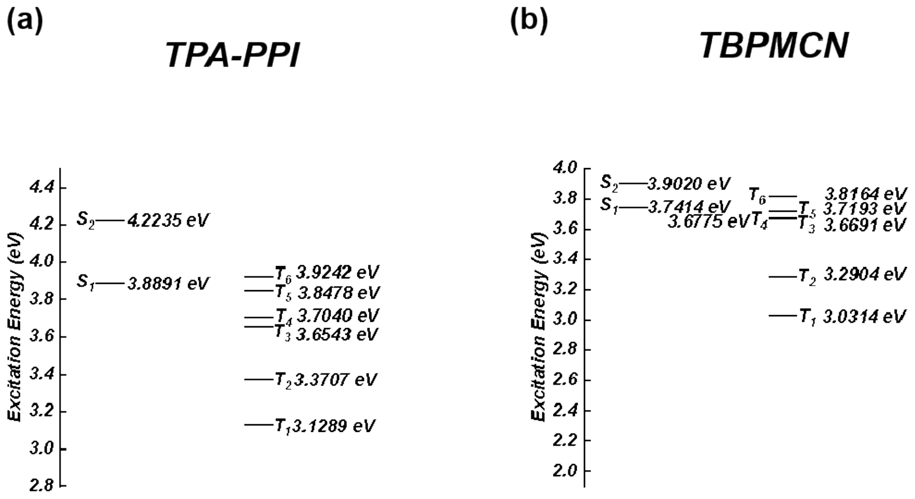

Figure S3. The Energy Level Diagram of (a) TPA-PPI and (b) TBPMCN.

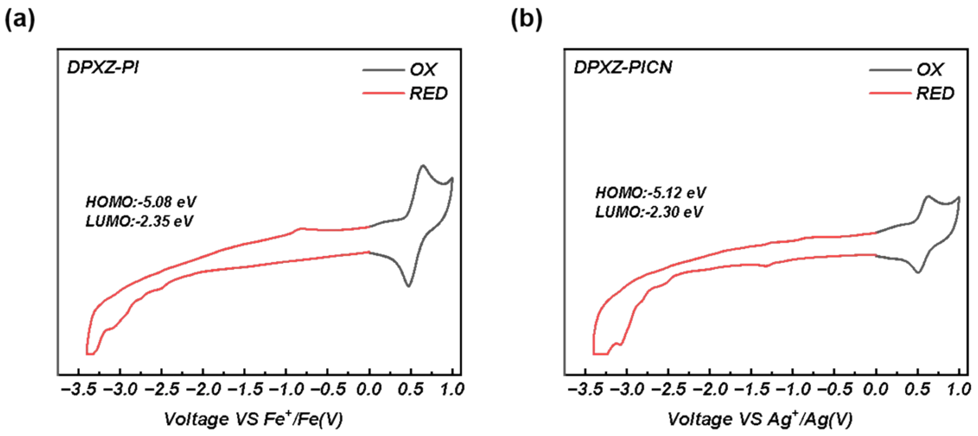

Figure S4. Cyclic voltammograms (CV) curves of (a) DPXZ-PI and (b) DPXZ-PICN.

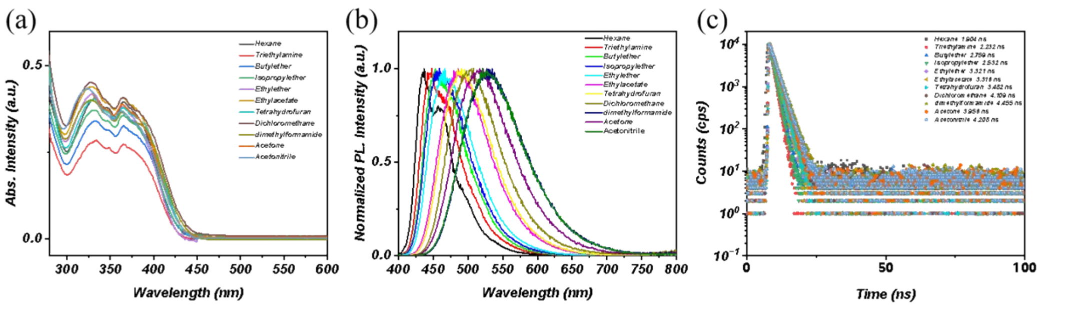

Figure S5. Photophysical properties of DPXZ-PI in different solvents. (a) Absorption spectra; (b) Emission spectra; (c) Lifetime.

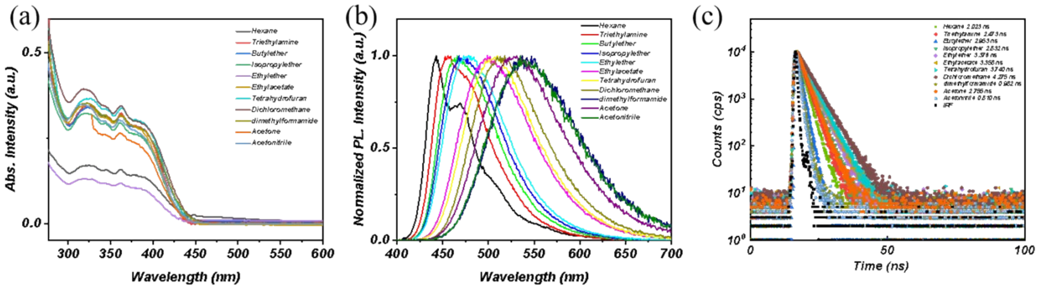

Figure S6. Photophysical properties of DPXZ-PICN in different solvents. (a) Absorption spectra; (b) Emission spectra; (c) Lifetime.

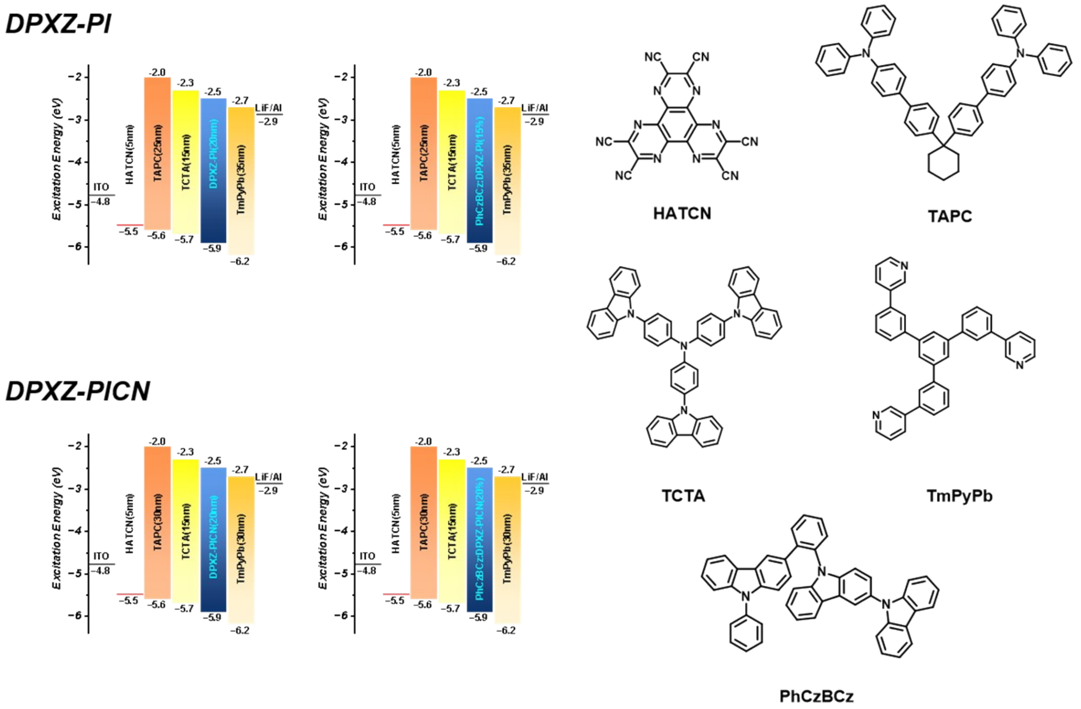

Figure S7. The OLED structure, the structures of the functional layers and the energy diagrams of the non-doped and doped OLEDs.

The non-doped OLED structure are ITO/HATCN(5nm)/TAPC(25nm)/TCTA(15nm)/DPXZ-PI (20nm)/TmPyPb(35nm)/LiF(1nm)/Al(100nm) and ITO/HATCN(5nm)/TAPC(30nm)/TCTA(15nm)/DPXZ-PICN(20nm)/TmPyPb(30nm)/LiF(1nm)/Al(100nm).

The doped OLED structure are ITO/HATCN(5nm)/TAPC(25nm)/TCTA(10nm)/PhCzBCz:DPXZ-PI/TmPyPb(35nm)/LiF(1nm)/Al(100nm) and ITO/HATCN(5nm)/TAPC(30nm)/TCTA(10nm)/PhCzBCz:DPXZ-PICN/TmPyPb(30nm)/LiF(1nm)/Al(100nm).

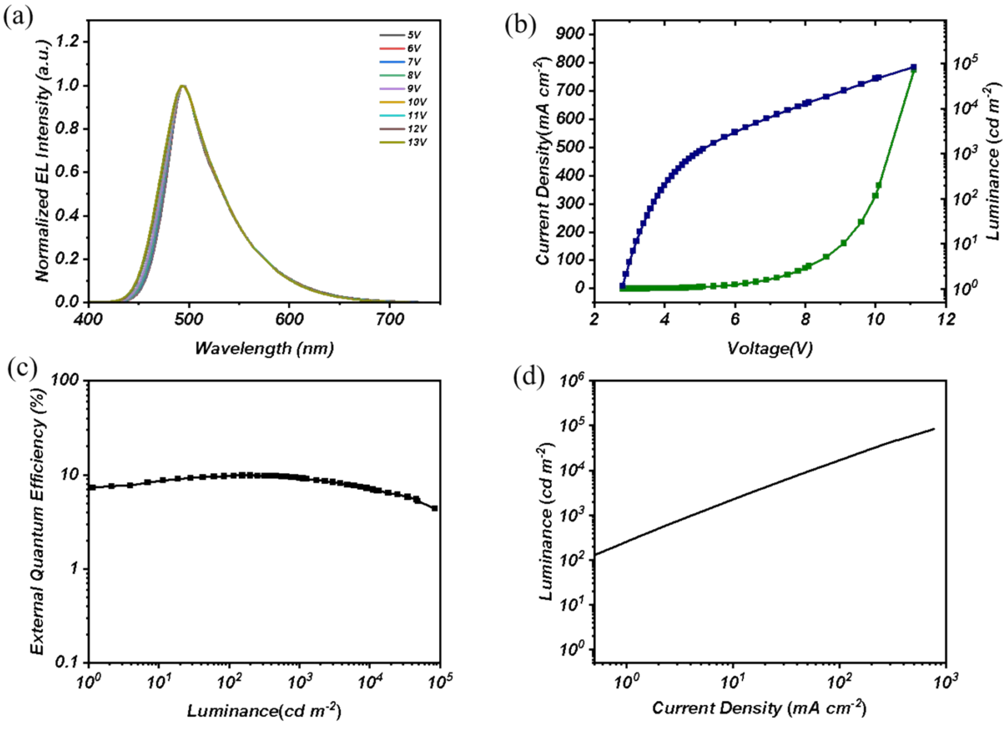

Figure S8. Non-doped OLED performance of DPXZ-PI. (a) Normalized EL spectra; (b) current density-voltage-Luminance curves; (c) EQE-Luminance curves; (d) Luminance-Current density curves.

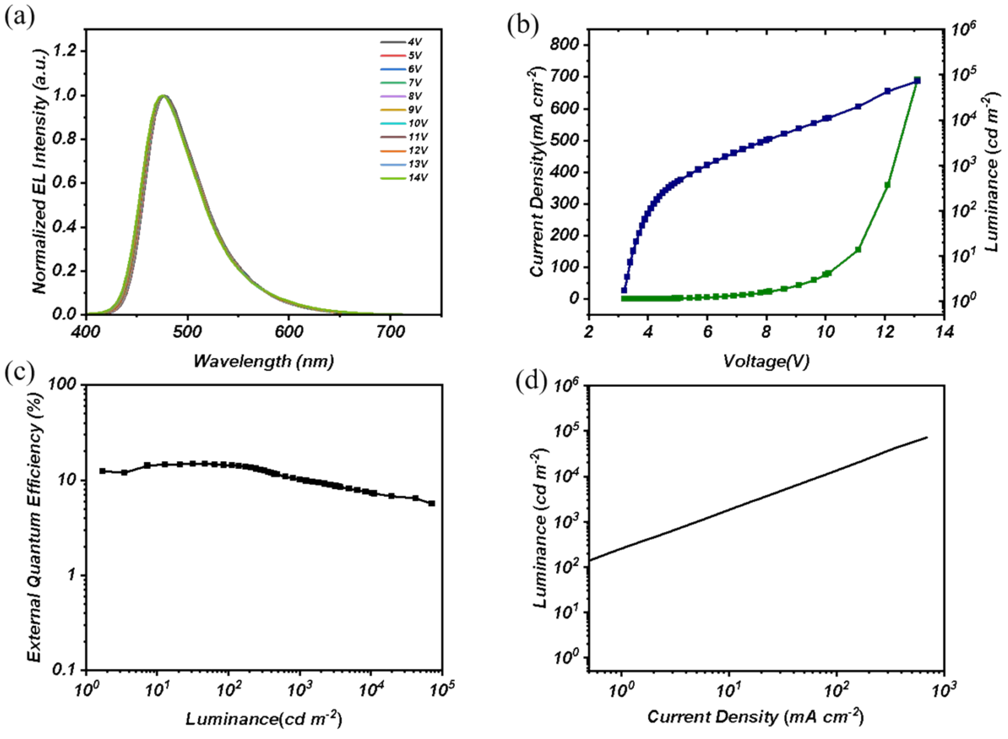

Figure S9. Doped OLED performance of DPXZ-PI. (a) Normalized EL spectra; (b) current density-voltage-Luminance curves; (c) EQE-Luminance curves; (d) luminance-Current density curves.

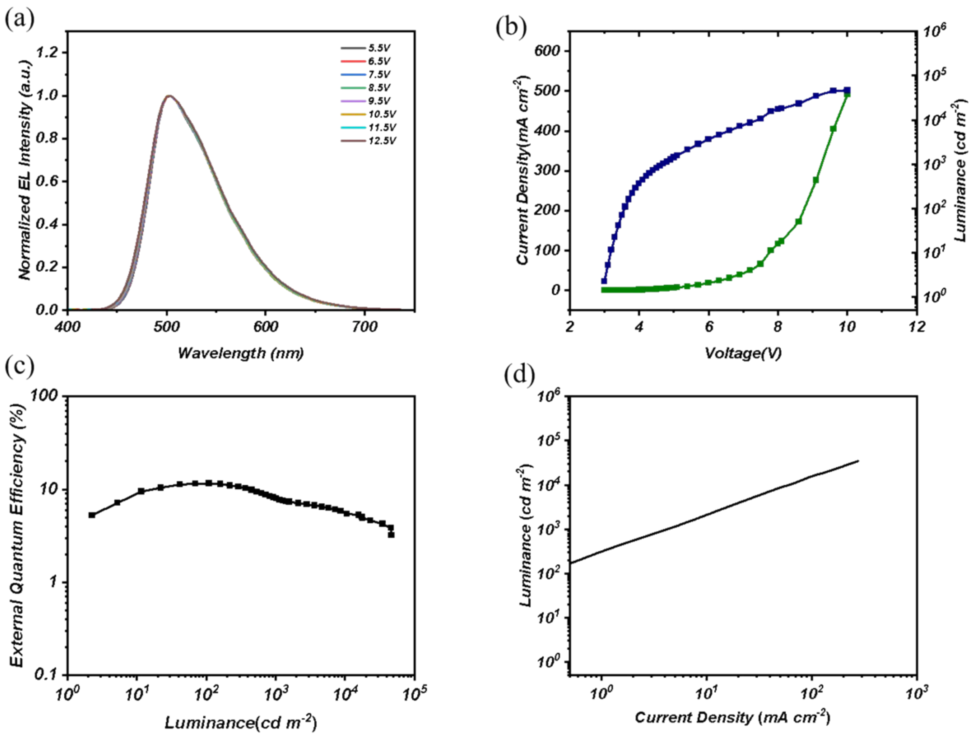

Figure S10. Non-doped OLED performance of DPXZ-PICN. (a) Normalized EL spectra; (b) current density-voltage-Luminance curves; (c) EQE-Luminance curves; (d) luminance-Current density curves.

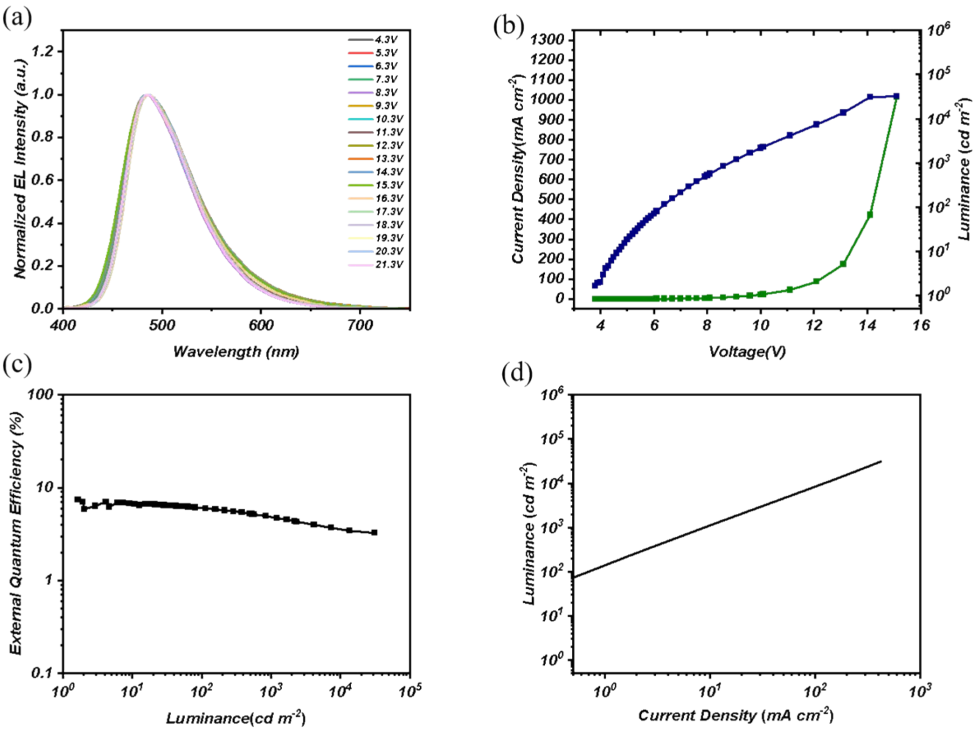

Figure S11. Doped OLED performance of DPXZ-PICN. (a) Normalized EL spectra; (b) current density-voltage-Luminance curves; (c) EQE-Luminance curves; (d) luminance-Current density curves.

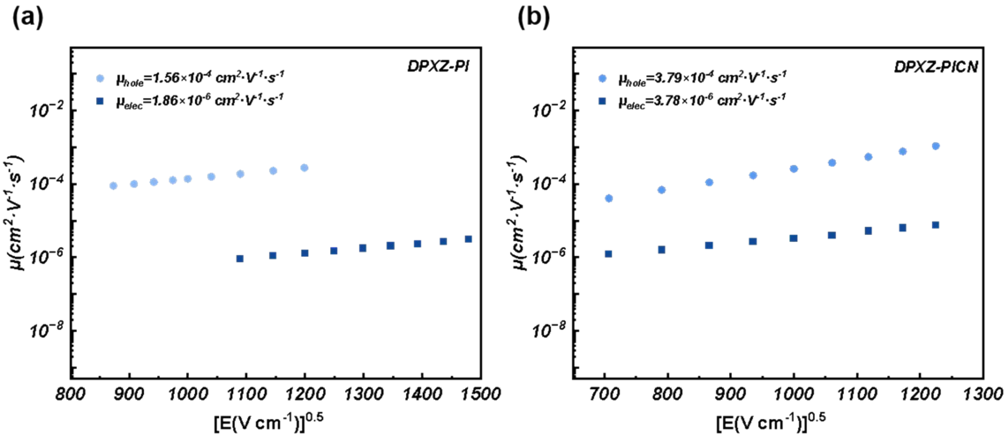

Figure S12. The carrier mobility of (a) DPXZ-PI and (b) DPXZ-PICN.

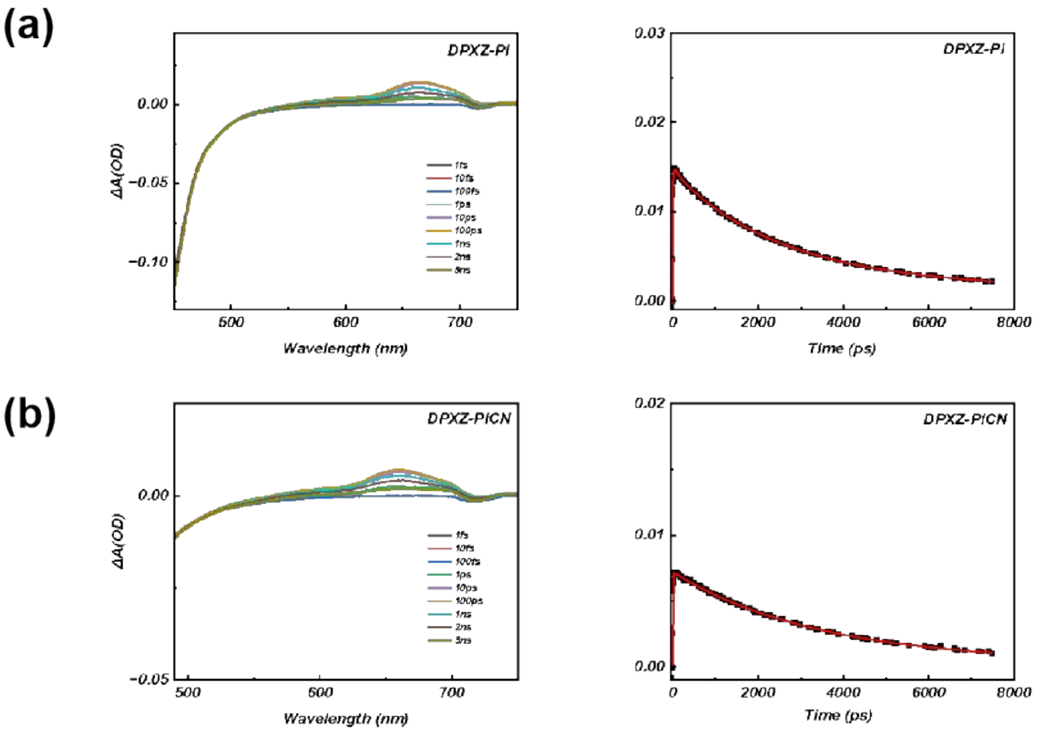

Figure S13. The ultrafast absorption and kinetics fitting of (a) DPXZ-PI and (b) DPXZ-PICN in visible region .

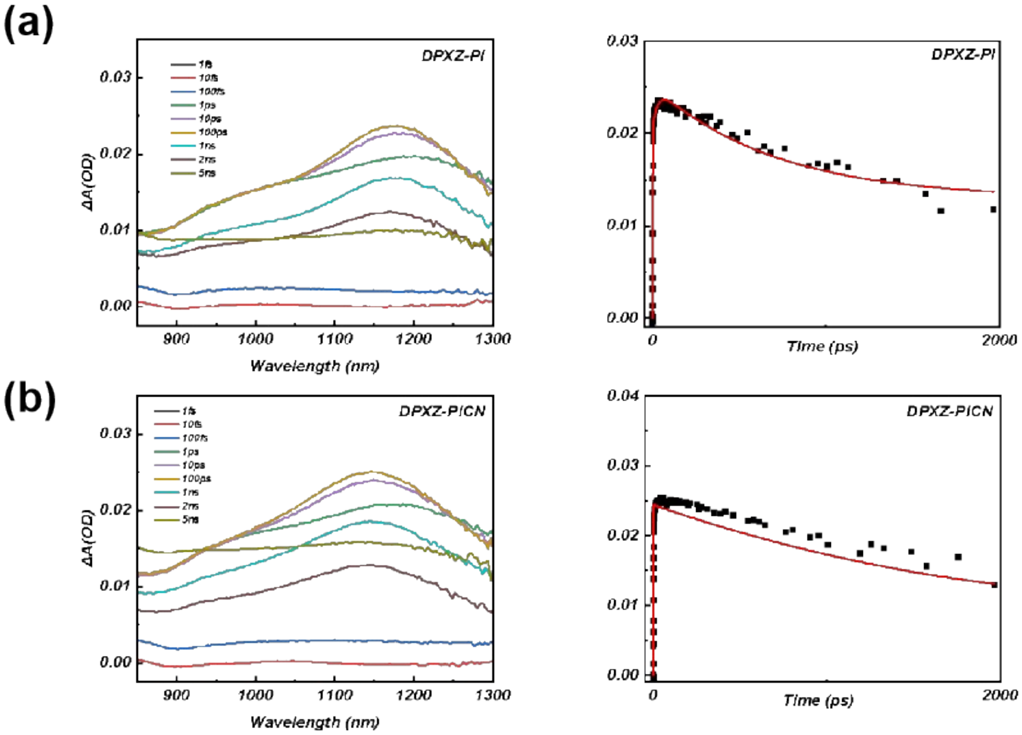

Figure S14. The ultrafast absorption and kinetics fitting of (a) DPXZ-PI and (b) DPXZ-PICN in

near-infrared region .

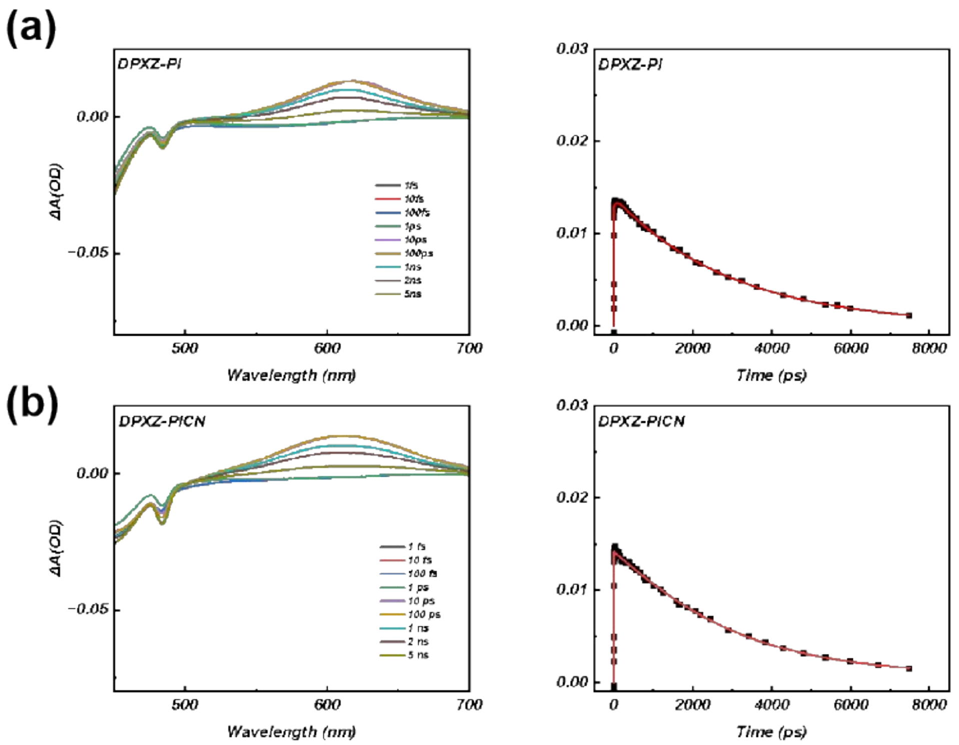

Figure S15. The ultrafast absorption and kinetics fitting of (a) DPXZ-PI and (b) DPXZ-PICN sensitized by $Ir(ppy)_3$ in visible region .

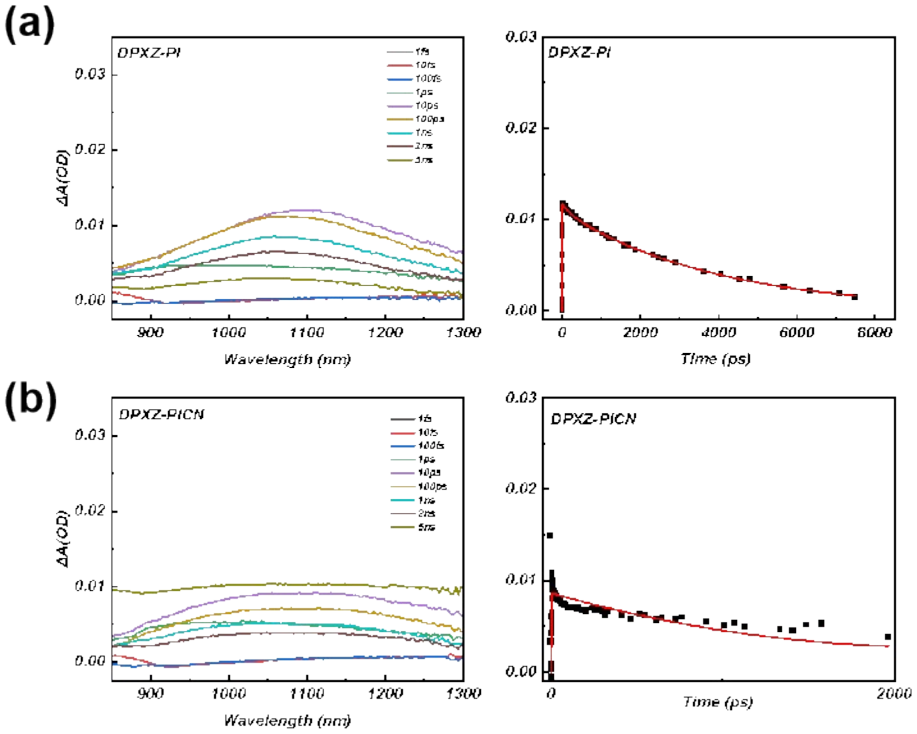

Figure S16. The ultrafast absorption and kinetics fitting of (a) DPXZ-PI and (b) DPXZ-PICN sensitized by Ir(ppy)$_3$ in near-infrared region .

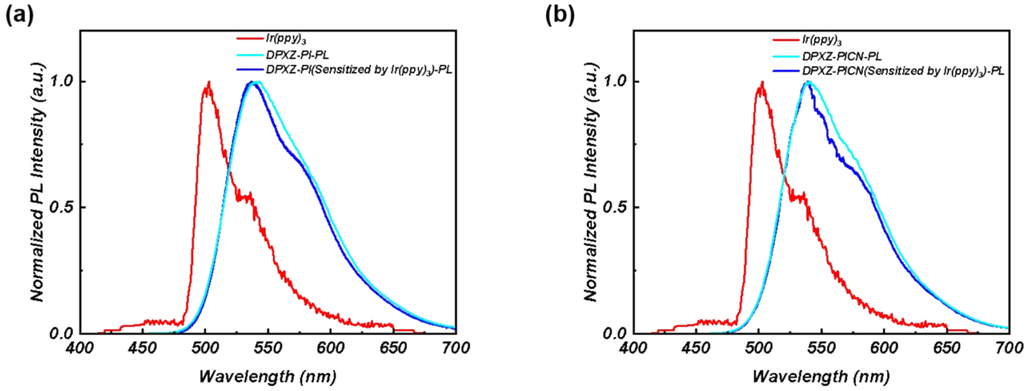

Figure S17. Phosphorescence spectra of (a) DPXZ-PI and (b) DPXZ-PICN before and after sensitization by Ir(ppy)$_3$.The concentration of the diluted solutions is 1×10^{-5} M.

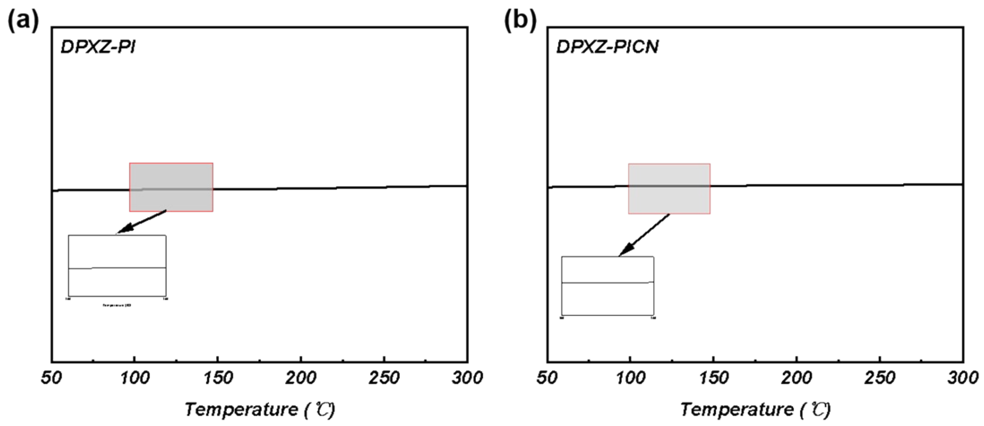

Figure S18. DSC of (a) DPXZ-PI and (b) DPXZ-PICN

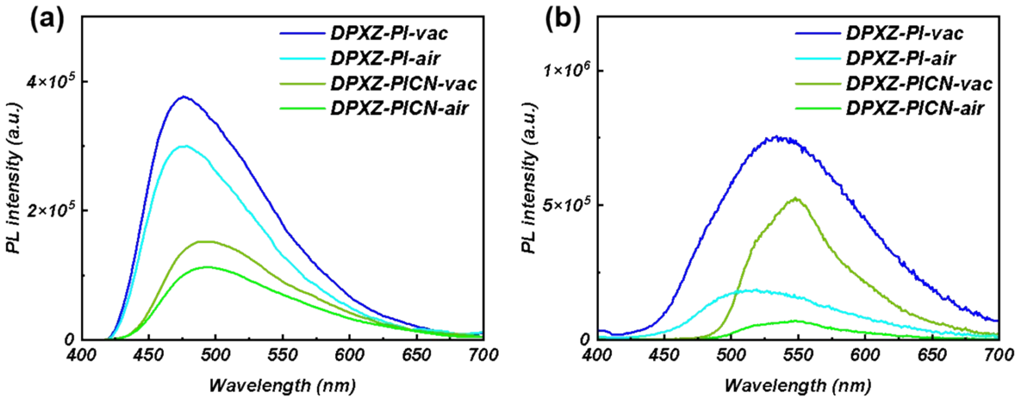

Figure S19. Comparison of emission spectra of DPXZ-PI and DPXZ-PICN in (a) PMMA-doped thin films and (b) powders under air and vacuum conditions

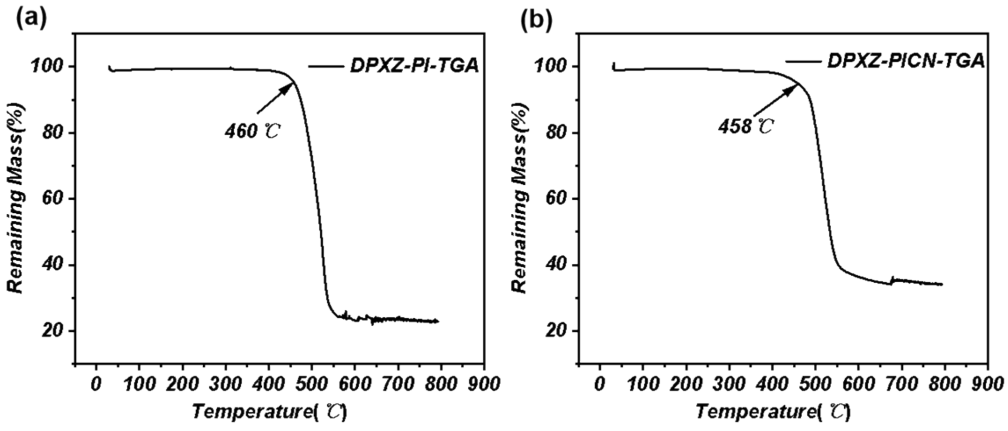

Figure S20. TGA of (a) DPXZ-PI and (b) DPXZ-PICN

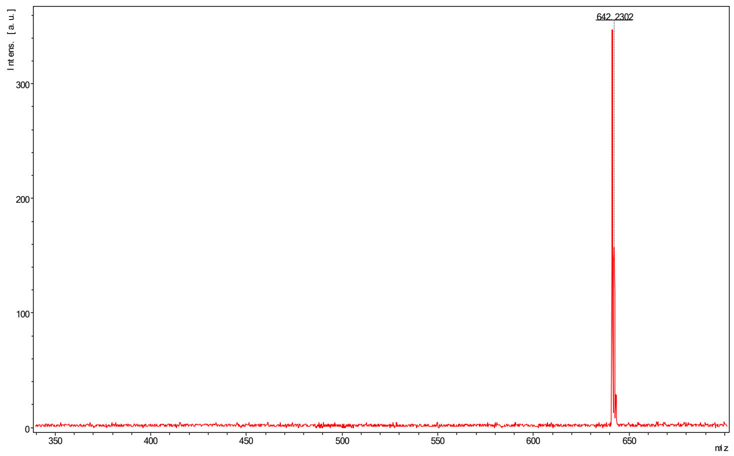

Figure S21. HRMS spectra of DPXZ-PI.

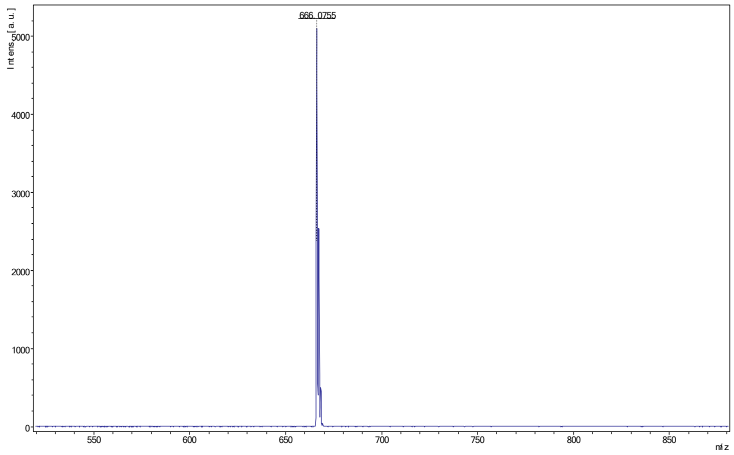

Figure S22. HRMS spectra of DPXZ-PICN.

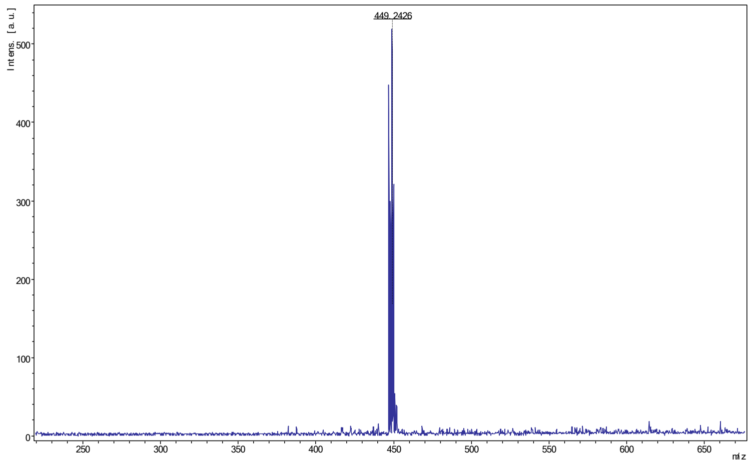

Figure S23. HRMS spectra of BrBPI.

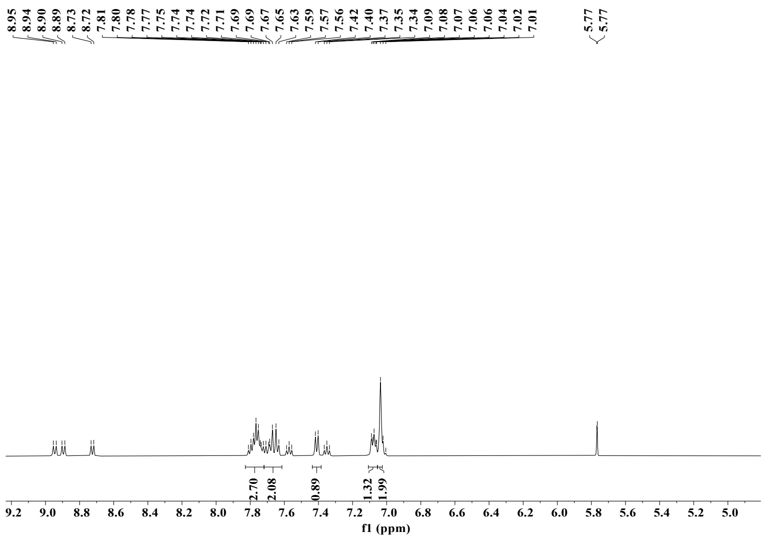

Figure S24. ^{1}H-NMR spectra of DPXZ-PI.

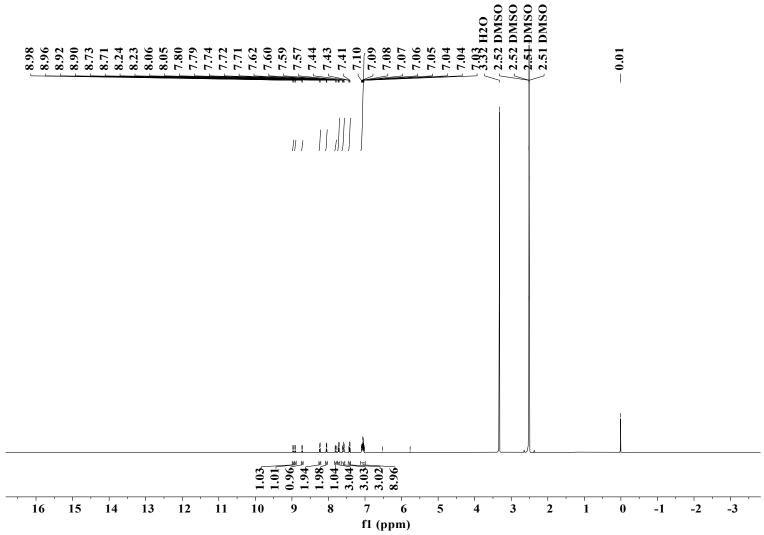

Figure S25. ^{1}H-NMR spectra of DPXZ-PICN.

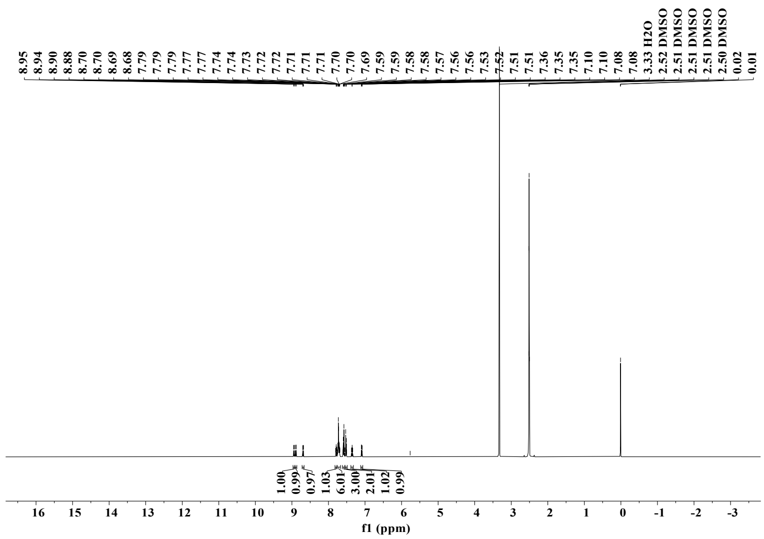

Figure S26. ^1H-NMR spectra of BrBPI.

Table S1. Proportion of excited-state CT components of DPXZ-PI and DPXZ-PICN.

		TBPMCN		DPXZ-PI		DPXZ-PICN	
		CT (%)	LE (%)	CT (%)	LE (%)	CT (%)	LE (%)
$S_0 \rightarrow S_1$	Co-axial direction	20.580	79.420	25.402	74.598	27.562	72.438
	Orthogonal direction	38.683	61.317	2.022	97.978	5.298	94.702

Table S2. The integral emission peak areas of DPXZ-PI and DPXZ-PICN in vacuum and air.

	Neat film in air	Neat film in vacuum	Doped film in air[a]	Doped film in vacuum[b]
DPXZ-PI	2.42624×10^{7}	1.05399×10^{8}	3.23955×10^{7}	4.17663×10^{7}
DPXZ-PICN	0.67186×10^{7}	4.6434×10^{7}	1.34872×10^{7}	1.78238×10^{7}

[a] [b]1% doped film in PMMA.

Table S3. The emission peaks in different solvents of DPXZ-PI.

Compound	Solvent	f	λ_{pl}[a](nm)	ν_f[b](cm^{-1})
	Hexane	0.001	437	22883.29
	Triethylamine	0.048	448	22321.43
	Butylether	0.097	453	22075.05
	Isopropylether	0.145	459	21786.49
	Ethylether	0.167	467	21413.27
DPXZ-PI	Ethylacetate	0.200	486	20576.13
	Tetrahydrofuran	0.210	491	20366.59
	Dichloromethane	0.217	508	19685.03
	Dimethylformamide	0.276	535	18691.58
	Acetone	0.284	517	19342.36
	Acetonitrile	0.305	521	19193.86

[a] emission peak [b] emission wavelength.

Table S4. The emission peaks in different solvents of DPXZ-PICN.

Compound	Solvent	f	λ_{pl}[a](nm)	ν_f[b](cm^{-1})
	Hexane	0.001	443	22573.36
	Triethylamine	0.048	456	21929.82
	Butylether	0.097	466	21459.23
	Isopropylether	0.145	468	21367.52
	Ethylether	0.167	478	20920.50
DPXZ-PICN	Ethylacetate	0.200	499	20040.08
	Tetrahydrofuran	0.210	510	19607.84
	Dichloromethane	0.217	518	19305.02
	Dimethylformamide	0.276	537	18621.97
	Acetone	0.284	531	18832.39
	Acetonitrile	0.305	535	18691.59

[a] emission peak [b] emission wavelength.

Table S5. The carrier mobilities of DPXZ-PI and DPXZ-PICN. The structure of hole-only device: ITO/HATCN (5 nm)/TAPC(20 nm)/DPXZ-PI(CN)(80 nm)/TAPC (20 nm)/LiF(1nm)/Al(100 nm). The structure of electron-only device :ITO/TmPyPb (20 nm)/DPXZ-PI(CN) (80 nm)/TmPyPb(20 nm)LiF (1 nm)/Al(100 nm).

Compound	Hole mobility[a][cm^2/(v·s)]	Electron mobility[b][cm^2/(v·s)]
DPXZ-PI	1.56×10^{-4}	1.86×10^{-6}
DPXZ-PICN	3.79×10^{-4}	3.78×10^{-6}

[a] [b]. Mobility under electric field of 4.0×10^5 V/cm.

3. References

[1] a) M. J. Frisch, G. W. Trucks, H. B. Schlegel, G. E. Scuseria, M. A. Robb, J. R. Cheeseman, G. Scalmani, V. Barone, G. A. Petersson, H. Nakatsuji, X. Li, M. Caricato, A. V. Marenich, J. Bloino, B. G. Janesko, R. Gomperts,

B. Mennucci, H. P. Hratchian, J. V. Ortiz, A. F. Izmaylov, J. L. Sonnenberg, Williams, F. Ding, F. Lipparini, F. Egidi, J. Goings, B. Peng, A. Petrone, T. Henderson, D. Ranasinghe, V. G. Zakrzewski, J. Gao, N. Rega, G. Zheng, W. Liang, M. Hada, M. Ehara, K. Toyota, R. Fukuda, J. Hasegawa, M. Ishida, T. Nakajima, Y. Honda, O. Kitao, H. Nakai, T. Vreven, K. Throssell, J. A. Montgomery Jr., J. E. Peralta, F. Ogliaro, M. J. Bearpark, J. J. Heyd, E. N. Brothers, K. N. Kudin, V. N. Staroverov, T. A. Keith, R. Kobayashi, J. Normand, K. Raghavachari, A. P. Rendell, J. C. Burant, S. S. Iyengar, J. Tomasi, M. Cossi, J. M. Millam, M. Klene, C. Adamo, R. Cammi, J. W. Ochterski, R. L. Martin, K. Morokuma, O. Farkas, J. B. Foresman, D. J. Fox, Wallingford, CT 2016; b) A. D. Becke, *The Journal of Chemical Physics* **1993**, 98, 5648.

[2] a) R. L. Martin, *The Journal of Chemical Physics* **2003**, 118, 4775; b) T. Lu, F. Chen, *Journal of Computational Chemistry* **2011**, 33, 580.

[3] W. Liu, F. Wang, L. Li, *Journal of Theoretical and Computational Chemistry* **2003**, 02, 257.
